# 3D hydrogels reveal medulloblastoma subgroup differences and identify extracellular matrix subtypes that predict patient outcome

**DOI:** 10.1002/path.5591

**Published:** 2020-12-17

**Authors:** Franziska Linke, Macha Aldighieri, Anbarasu Lourdusamy, Anna M Grabowska, Snow Stolnik, Ian D Kerr, Catherine LR Merry, Beth Coyle

**Affiliations:** ^1^ Children's Brain Tumour Research Centre, School of Medicine Biodiscovery Institute, University of Nottingham Nottingham UK; ^2^ Division of Cancer and Stem Cells, School of Medicine Biodiscovery Institute, University of Nottingham Nottingham UK; ^3^ Division of Molecular Therapeutics and Formulation, School of Pharmacy University of Nottingham Nottingham UK; ^4^ School of Life Sciences University of Nottingham Nottingham UK

**Keywords:** medulloblastoma, laminin, vitronectin, subtypes, hydrogel, ECM, metastasis, chemoresistance, 3D model

## Abstract

Medulloblastoma (MB) is the most common malignant brain tumour in children and is subdivided into four subgroups: WNT, SHH, Group 3, and Group 4. These molecular subgroups differ in their metastasis patterns and related prognosis rates. Conventional 2D cell culture methods fail to recapitulate these clinical differences. Realistic 3D models of the cerebellum are therefore necessary to investigate subgroup‐specific functional differences and their role in metastasis and chemoresistance. A major component of the brain extracellular matrix (ECM) is the glycosaminoglycan hyaluronan. MB cell lines encapsulated in hyaluronan hydrogels grew as tumour nodules, with Group 3 and Group 4 cell lines displaying clinically characteristic laminar metastatic patterns and levels of chemoresistance. The glycoproteins, laminin and vitronectin, were identified as subgroup‐specific, tumour‐secreted ECM factors. Gels of higher complexity, formed by incorporation of laminin or vitronectin, revealed subgroup‐specific adhesion and growth patterns closely mimicking clinical phenotypes. ECM subtypes, defined by relative levels of laminin and vitronectin expression in patient tissue microarrays and gene expression data sets, were able to identify novel high‐risk MB patient subgroups and predict overall survival. Our hyaluronan model system has therefore allowed us to functionally characterize the interaction between different MB subtypes and their environment. It highlights the prognostic and pathological role of specific ECM factors and enables preclinical development of subgroup‐specific therapies. © 2020 The Authors. *The Journal of Pathology* published by John Wiley & Sons, Ltd. on behalf of The Pathological Society of Great Britain and Ireland.

## Introduction

Medulloblastoma (MB) is the most common malignant brain tumour in children and is subdivided into four subgroups: WNT, sonic hedgehog (SHH), Group 3, and Group 4. These molecular subgroups are characterized by different metastasis patterns and related prognosis rates [1, 2, 3, 4]. During the past decade, these MB subgroups have been defined based on gene expression, and further refined with the help of additional DNA methylation data, resulting in four major MB subgroups with additional subtypes [1, 2, 5, 6]. The introduction of MB subgroups added a new classification tool and facilitated the differentiation of low‐, average‐, high‐, and very high‐risk patients [7, 8] as an indicator of outcome.

Metastatic dissemination is the most powerful predictor of poor outcome across all MB subgroups [9, 10, 11]. Recently, Zapotocky *et al* observed and quantified characteristic tumour phenotypes according to the MB subgroups as radiological differences in primary tumour sizes and metastatic phenotypes [12, 13]. In metastatic MB, Group 3 primary tumours are usually significantly smaller than SHH or Group 4 tumours; hence they hypothesized that an earlier dissemination event might be a characteristic of Group 3 tumours. Importantly, they also categorized the type of metastasis and found that Group 3 tumours predominantly spread as nodules and a thin laminar coating (laminar phenotype). In contrast, SHH tumours predominantly metastasize as nodules only (nodular phenotype), while Group 4 tumours can metastasize as nodules as well as a laminar coating [13]. The majority of genomic and transcriptional alterations that have been identified to date have, however, not been linked to signalling aberrations that could explain leptomeningeal spread or be targeted in metastatic MB subgroups [14].

Based on the above observations, we hypothesized that interaction with the surrounding extracellular matrix (ECM) might differ between the MB subgroups, thus explaining subgroup‐specific adhesion, invasion, and growth patterns. The radiological findings suggest that the most significant differences in ECM interaction and remodelling might be expected between Group 3 and SHH tumours, while Group 4 tumours would be characterized by an intermediate phenotype. Investigation of subgroup‐specific ECM interactions required a modifiable but realistic *in vitro* model system that allows long‐term growth and remodelling of the local extracellular matrix by encapsulated tumour cells. The brain microenvironment is unique in its composition and characteristics [15, 16, 17]. In contrast to the ECM environment in many other organs of the human body, fibrous proteins such as collagens are in low abundance, whereas the glycosaminoglycan hyaluronan (HA) and HA‐proteoglycan superstructures are major components of the brain ECM [17, 18]. No *in vitro* system currently exists that addresses the unique composition and characteristics of the brain microenvironment.

Here, we developed a 3D cell culture model that allows investigation of the different metastatic tumour phenotypes observed in medulloblastoma subtypes. We used a 3D HA hydrogel model to show that the specific ECM composition as well as active ECM remodelling by MB tumour cells alters the adhesion, invasion, and growth characteristics of the tumour. Our model retains cell–HA interactions, which are potentially a crucial part of the brain tumour–matrix interface [19, 20]. Based on the RNA and protein expression levels of MB subgroup‐specific ECM components and receptors, we identified the glycoproteins laminin and vitronectin as functional markers that can act as predictors of a laminar or nodular phenotype. The resultant ECM subtypes correlated with overall patient survival and highlight MB subgroup differences in ECM interaction.

## Materials and methods

### HA hydrogel preparation

Hyaluronan cross‐linked hydrogels were prepared according to the manufacturer's recommendations (HyStem; BioTime Inc, Alameda, CA, USA) using particular hyaluronan (1%) and Extralink (2% PEGDA) concentrations. Further details can be found in Supplementary materials and methods.

### Cell viability assay

For cell viability assays, Prestoblue reagent (Thermo Fisher, Waltham, MA, USA) was used according to the manufacturer's instructions and fluorescence intensity was measured after 40 min incubation time using a microplate reader (FLOUstar Omega; BMG Labtech, Ortenberg, Germany). For 3D hydrogel viability assays, all gels were washed four times with HBSS buffer (Thermo Fisher) before fresh medium was added to each well. Cell viability within each gel was measured weekly after drug or vehicle treatment.

### Vincristine drug treatment in 2D and 3D

Vincristine (VCR) (S1241; Selleckchem, Munich, Germany; 825 Da) was added to the cell culture medium at three different concentration of VCR dissolved in DMSO (Sigma‐Aldrich, St Louis, MO, USA). The VCR concentrations chosen were the IC_50_ of a single VCR dosage (5 nm) as well as the two‐fold (10 nm) and ten‐fold IC_50_ (50 nm) as defined in spheroid cultures. DMSO was used as a vehicle control. For the 2D experiment, VCR was renewed 24 h after the first treatment. For the 3D gel experiment, 3‐week‐old hydrogels were treated with VCR and VCR renewal was performed 24, 72, and 144 h after the first dose. In order to assess the cell's recovery potential, all gels were washed and covered with fresh, drug‐free medium 1 week after the final VCR treatment and monitored for a further 4 weeks (total experiment time: 8 weeks).

### Statistics

Results are shown as mean ± SEM of the indicated number of independent experiments. The statistical significance of differences of group results was compared using one‐ or two‐way analysis of variance (ANOVA) with a Dunnett's *post hoc* test to correct for multiple comparisons as indicated. Normal distribution and homogeneity of variance were tested using the Kolmogorov–Smirnov test and the *F*‐test. For nonparametric group results, the Kruskal–Wallis test with Dunn's *post hoc* test was performed. Significance levels are indicated as *p < 0.05, **p < 0.01, and ***p < 0.001. All statistical analyses and plots were carried out using GraphPad Prism 7.05 (GraphPad Software Inc, La Jolla, CA, USA) unless stated otherwise. The number of biological samples, corresponding statistical test, and significance levels are indicated in each figure legend. Kaplan–Meier survival data analysis was performed using the R2: Genomics Analysis and Visualization Platform (http://r2.amc.nl) and statistical significance was tested using the log‐rank test as described by Bewick *et al* [21].

Detailed methods for Medulloblastoma cell lines, culturing, and reagents; HA hydrogel preparation, long‐term cell culture, and quantification of invasion; Etoposide drug treatment in 3D; Western blotting; RNA isolation and RNA sequencing; RNA sequence data analysis; Microarray data analysis; Combined data analysis; Immunohistochemistry of HA hydrogels and tissue microarrays; and ECM protein enrichment analysis, ECM subtype definition, and overall survival analysis are presented in Supplementary materials and methods.

## Results

### Long‐term 3D hydrogel culture recapitulates MB subgroup‐specific invasion patterns and chemotherapy resistance

In our study we used six different medulloblastoma cell lines representing the SHH, Group 3, and Group 4 medulloblastoma subgroups as validated by RNA sequencing (supplementary material, Figure S1). To study growth differences between MB subgroups and the role of the ECM, we compared long‐term growth of MB cell lines encapsulated in hyaluronan hydrogels with conventional 2D cell culture. In 2D, cell growth does not reflect the clinical phenotype or aggressiveness of the particular subgroup (supplementary material, Figure S2A). For example, Group 4 cell lines show the slowest growth *in vitro* (supplementary material, Figure S2A: CHLA‐01/01R‐MED), although Group 4 MB is characterized by high metastasis rates and intermediate to poor prognosis concordant with high tumour aggressiveness compared with the less aggressive p53 wild‐type SHH subtype (ONS76).

We hypothesized that a more realistic brain‐like environment would uncover MB subgroup‐specific phenotypes and allow us to investigate functionally relevant interactions between the tumour cells and the ECM. Since the brain ECM is relatively rich in glycosaminoglycans compared to fibrous proteins such as collagens, we decided to use modifiable HA hydrogels as a simple 3D model. MB cell lines were encapsulated as single cells or small clusters (Group 4) in HA hydrogels and their growth was monitored for up to 60 days (supplementary material, Figure S2B–F). The HA hydrogels supported the growth of nodules containing actively dividing cells for at least 60 days.

**Figure 1:**
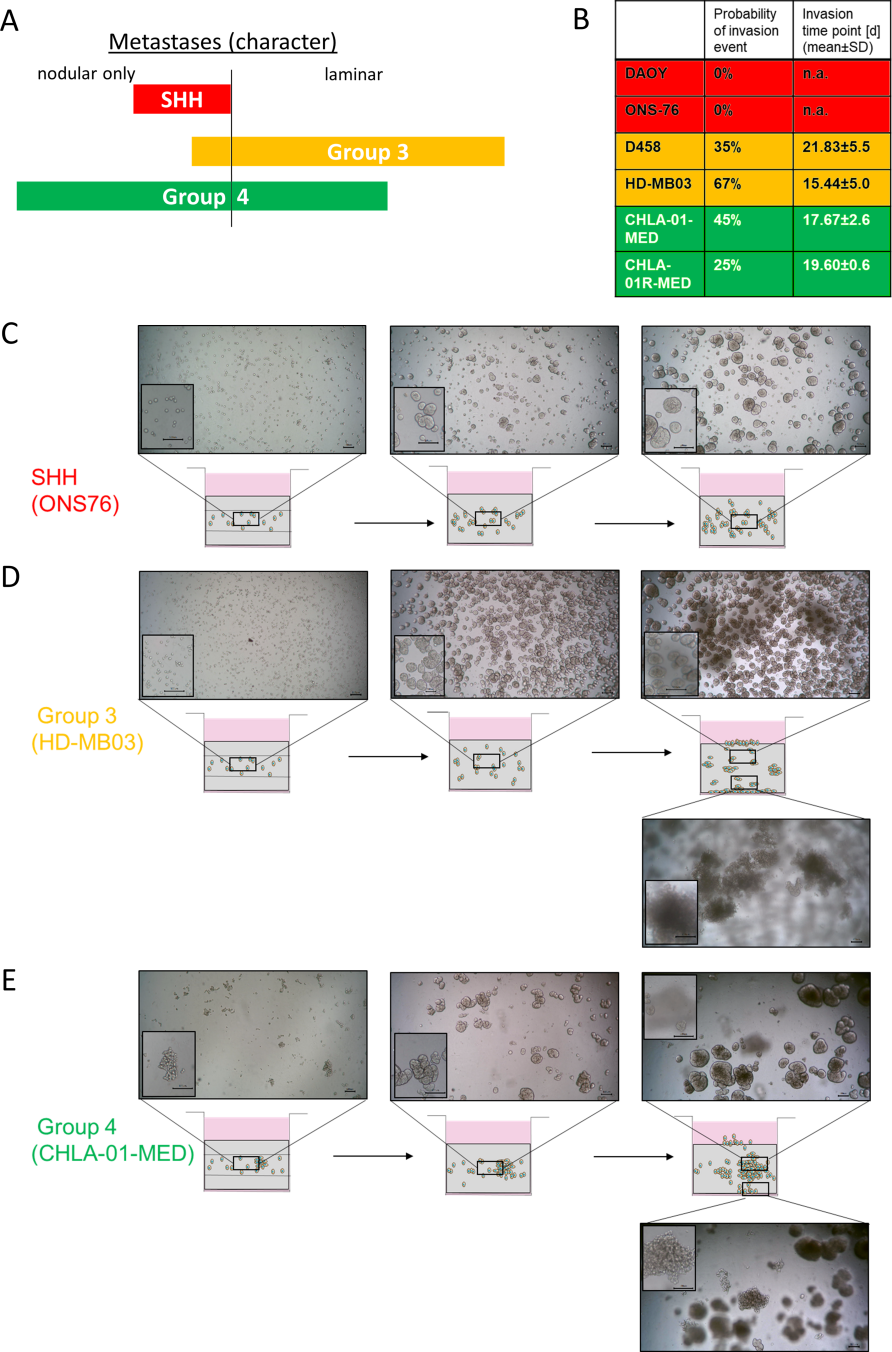
Clinically relevant subgroup‐specific invasion patterns of Group 3 and Group 4 MB cell lines can be observed after 2–3 weeks of long‐term growth in the HA hydrogels. (A) Metastatic character differs between MB subgroups according to Zapotocky *et al* [13]. Group 3 tumours predominantly metastasize as a laminar coating, while SHH tumours predominately metastasize as nodules and Group 4 tumours have an intermediate phenotype. (B) During long‐term 3D culture, Group 3 and Group 4 cell lines frequently invade through the hydrogel and grow as an additional monolayer either on the surface or on the bottom of the well, while this was never observed using SHH cell lines (*n* = 34; DAOY, D458; *n* = 24 HD‐MB03; *n* = 22; ONS76, CHLA‐01‐MED; *n* = 20 CHLA‐01R‐MED). Representative images of the invasive behaviour over time are shown for the SHH line ONS76 (C), the Group 3 cell line HD‐MB03 (D), and the Group 4 cell line CHLA‐01‐MED (E) (scale bar = 100 μm). Images are of the areas indicated in the schematic, with the insets representing different focal planes to delineate nodular and laminar growth phenotypes.

Radiological studies [13] have shown that metastasis patterns differ between the MB subgroups. While SHH tumours are predominantly nodular, the more aggressive Group 3 tumours metastasize predominantly in a laminar phenotype which is defined by the occurrence of nodules and additional thin laminar coatings (Figure 1A). Group 4 tumours are intermediate, with either a laminar or a nodular phenotype (Figure 1A). Long‐term growth of MB cell lines as nodules inside HA hydrogels also reveals subgroup‐specific invasion patterns and time courses. Since the gels are prepared as ‘sandwich layers’ (see the Materials and methods section), a defined cell‐free gel volume has to be crossed to reach the gel–media interface or the well bottom. Some cell lines were found to invade through the gels and form a monolayer on the bottom or on the gel surface in addition to the nodules in the centre. The occurrence and time point of these invasion events differ between the MB subgroups (Figure 1B). While neither SHH cell line invaded through the HA hydrogels (Figure 1B,C), Group 3 and Group 4 cell lines frequently invaded after 2–3 weeks and formed the additional laminar coating (Figure 1B,D,E). The cell line‐specific invasion in the HA hydrogels correlates well with the described radiological observations and underlines the benefits of the 3D hydrogel model to demonstrate subgroup‐specific intrinsic invasion capacities.

**Figure 2:**
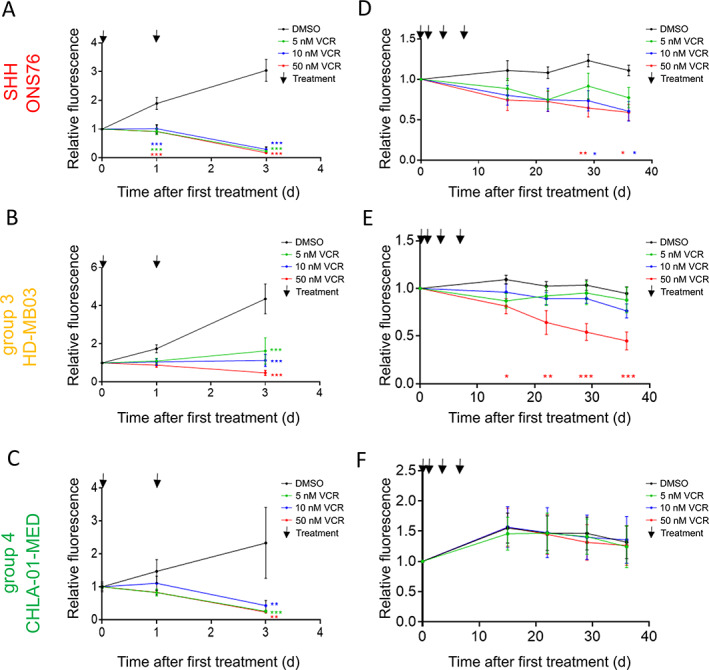
Vincristine (VCR) treatment kills MB cell lines effectively in 2D culture, while Group 3 nodules growing in HA hydrogels display strong resistance comparable to clinical observations. The VCR concentrations chosen were the IC_50_ of a single VCR dosage (5 nm) as well as the two‐fold (10 nm) and ten‐fold IC_50_ (50 nm). In 2D cell culture, 5 nm, 10 nm, and 50 nm VCR significantly reduces the viability of (A) the SHH cell line ONS76, (B) the Group 3 cell line HD‐MB03, and (C) the Group 4 cell line CHLA‐01‐MED (mean ± SEM, *n* = 3 ONS76; *n* = 4 HD‐MB03; *n* = 3 CHLA‐01‐MED; two‐way ANOVA and Dunnett's *post hoc* test). After 3 weeks of growth inside the HA hydrogels, (D) the SHH cell line ONS76, (E) the Group 3 cell line HD‐MB03, and (F) the Group 4 cell line CHLA‐01‐MED were treated four times with either 5 nm, 10 nm or 50 nm VCR or vehicle during 1 week and cell viability was monitored for the following 4 weeks in the absence of drug/vehicle. While 10 nm and 50 nm VCR significantly reduce cell viability in the SHH cell line, only 50 nm VCR significantly affected Group 3 cell viability. The Group 4 cell line was completely resistant to VCR in 3D (mean ± SEM, *n* = 3; two‐way ANOVA and Dunnett's *post hoc* test). *p < 0.05, **p < 0.01, and ***p < 0.001.

We compared the response of MB nodules and cells grown in 2D *in vitro* assays with a chemotherapeutic agent. Vincristine (VCR) is commonly used for MB treatment, for example as part of the Packer protocol [22] but also in ongoing trials (NCT01878617, NCT02724579, NCT02066220), and is usually given in repeated doses. For the 2D experiments, MB cell lines were treated twice within 3 days with one of three different VCR concentrations, and cell viability was monitored. All MB cell lines were killed with the repeated VCR dosage regardless of the concentration applied (Figure 2A–C). For the 3D hydrogel experiment, the cell lines were embedded inside the gels and allowed to form and establish nodules for 3 weeks before treatment. Over the following week, four treatments of VCR were given, and nodule viability was monitored for a further 4 weeks. The IC_50_ VCR concentration did not significantly reduce cell viability of the SHH cell line but the two‐fold and ten‐fold VCR IC_50_ were effective (Figure 2D). In contrast, the highly aggressive Group 3 and Group 4 cell lines remained almost unaffected by the repeated treatment with one‐ and two‐fold VCR IC_50_ concentrations. Only the ten‐fold VCR IC_50_ significantly reduced cell viability of the Group 3 cell line, although it still had no effect on the Group 4 cell line (Figure 2E,F). These subgroup‐specific drug responses could not have been predicted from the 2D *in vitro* data but are in line with clinical observations demonstrating the efficacy of chemotherapy for WNT/SHH subgroups but not Group 3/4 MB patients [23].

To further evaluate the subgroup‐specific response to chemotherapy, we treated 3‐week‐old hydrogels with etoposide (ETO; four treatments in 1 week) and monitored nodular growth for several weeks (supplementary material, Figure S3A,B). While the viability of the SHH cell line decreased depending on the ETO dosage (supplementary material, Figure S3A), the Group 3 nodules remained unaffected by 0.5 μm and 1 μm ETO treatments (supplementary material, Figure S3B). Only the ten‐fold ETO IC_50_ effectively decreased Group 3 cell viability.

Taken together, our data show that the 3D HA hydrogel is a relevant model for MB that can recapitulate clinically distinct characteristics of MB subgroups and therefore could be used to investigate specific differences in MB subgroup–ECM interactions.

### The glycoproteins laminin and vitronectin identify MB ECM subgroups

Next, we aimed to identify subgroup‐specific ECM components. To identify important and specific ECM markers for the MB subgroups, we used the largest publicly available MB gene expression data set [6]. We analysed ECM‐associated genes with highest expression in Group 3, intermediate expression in Group 4, and lowest expression in SHH tumours, and vice versa (Materials and methods section and supplementary material, Figure S4). Vitronectin (*VTN*) was the most highly expressed gene in Group 3 tumours relative to SHH and showed intermediate expression in Group 4 (supplementary material, Figures S4A–D and S5A). Seven ECM component encoding genes with the opposite expression pattern, i.e. highest expression in SHH tumours, were identified in the same way (Materials and methods section and supplementary material, Figure S4A–C,E). These included *LAMA1* (encoding the α subunit of laminin 111), *ITGA6*, and *ITGA7* (supplementary material, Figures S4E and S5B). Laminins are heterotrimeric proteins that consist of α, β, and γ subunits; brain basement membranes include laminin isoforms 111 and the homologous isoform 211, in contrast to the endothelial laminins 411 and 511 [24]. We therefore compared additionally the gene expression of β and γ subunits. We found α2 and γ1 subunits to be significantly higher‐expressed in SHH tumours, whereas Group 3 and Group 4 tumours expressed equivalent levels (supplementary material, Figure S4A–C). In addition, ITGA6 is relevant for binding laminin‐111 and ‐211, while ITGA7 is more relevant for laminin‐211, further supporting the role of laminin‐111 and ‐211 in SHH tumours.

**Figure 3:**
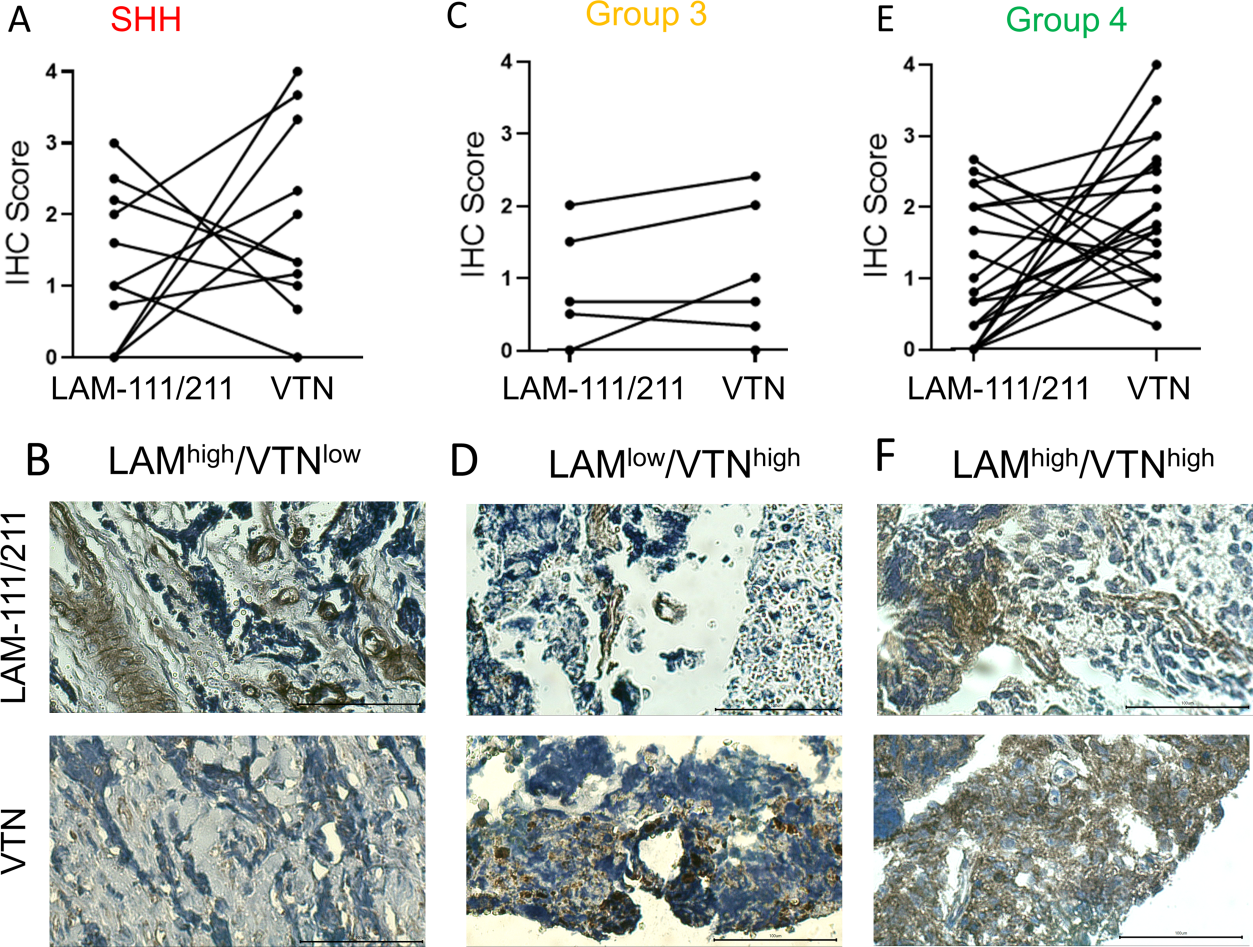
Protein expression of laminin and vitronectin in patients' TMAs defines relevant ECM subgroups in MB. (A) IHC staining of SHH tumours (*n* = 11) reveals two main ECM patterns (LAM^high^/VTN^low^and LAM^low^/VTN^high^). (B) An example of a ‘LAM^high^/VTN^low^’ SHH tumour with low vitronectin positivity. (C) In Group 3 tumours (*n* = 6), the predominant expression pattern is equivalent expression of laminin and vitronectin next to the ‘LAM^low^/VTN^high^’ pattern. (D) An example of a ‘LAM^low^/VTN^high^’ expressing tumour shows moderate to high staining for vitronectin and low laminin expression. (E) For MB Group 4 (*n* = 25), ‘LAM^high^/VTN^low^’ and ‘LAM^low^/VTN^high^’ groups are more common than the equivalent expression pattern of laminin and vitronectin. (F) An example for a ‘LAM^high^/VTN^high^’ Group 4 tumour is shown (scale bar = 100 μm). Examples for the other expression patterns are shown in supplementary material, Figure S5 and clinical information is listed in supplementary material, Table S1.

We concluded that laminin‐111/211 and vitronectin can be used as initial candidates for investigating MB subgroup–specific ECM interactions. It is, however, well described that matrix deposition/manipulation cannot necessarily be predicted from gene expression data [25]. Therefore, we stained MB tissue microarray sections for proteins of the identified gene markers (TMA; supplementary material, Table S1). Interestingly, we identified different combinations of laminin‐111/211 and vitronectin expression patterns in the MB subgroups (Figure 3). In SHH tumours, we found LAM^high^/VTN^low^ samples with high laminin‐111/211 expression but low vitronectin protein levels (Figure 3A,B) as well as samples with high vitronectin but low LAM‐111/211 (Figure 3A; supplementary material, Figure S6A). For Group 3 and Group 4 tumours, as well as the LAM^high^/VTN^low^ and LAM^low^/VTN^high^ pattern (Figure 3C,D; supplementary material, Figure S6B,D,E), two additional patterns of equivalent low or high expression of both markers were identified (Figure 3E,F; supplementary material, Figure S6C). In the majority of cases, however, high expression of both glycoproteins, laminin‐111/211 and vitronectin, seemed to be mutually exclusive, suggesting opposing roles of laminin and vitronectin, which is in agreement with the gene expression data. Indeed, these ECM patterns defined by laminin and vitronectin protein expression on patient's TMAs could also be seen at the gene expression level using the data set from Cavalli *et al* [6] (supplementary material, Figure S7). Laminin and vitronectin gene expression in the Cavalli data set (supplementary material, Figures S4, S5, and S7) and protein expression in the TMAs (Figure 3) show that there are different combinations of LAM/VTN (high/low, low/low, high/high, low/high) that present to varying degrees in the MB subgroups, indicating that the ECM is a key candidate for the different tumour phenotypes regarding adhesion, invasion, and migration.

### Laminin and vitronectin incorporation into hyaluronan gels induces nodular or laminar growth patterns; laminin and vitronectin are also actively secreted by the cell lines

**Figure 4:**
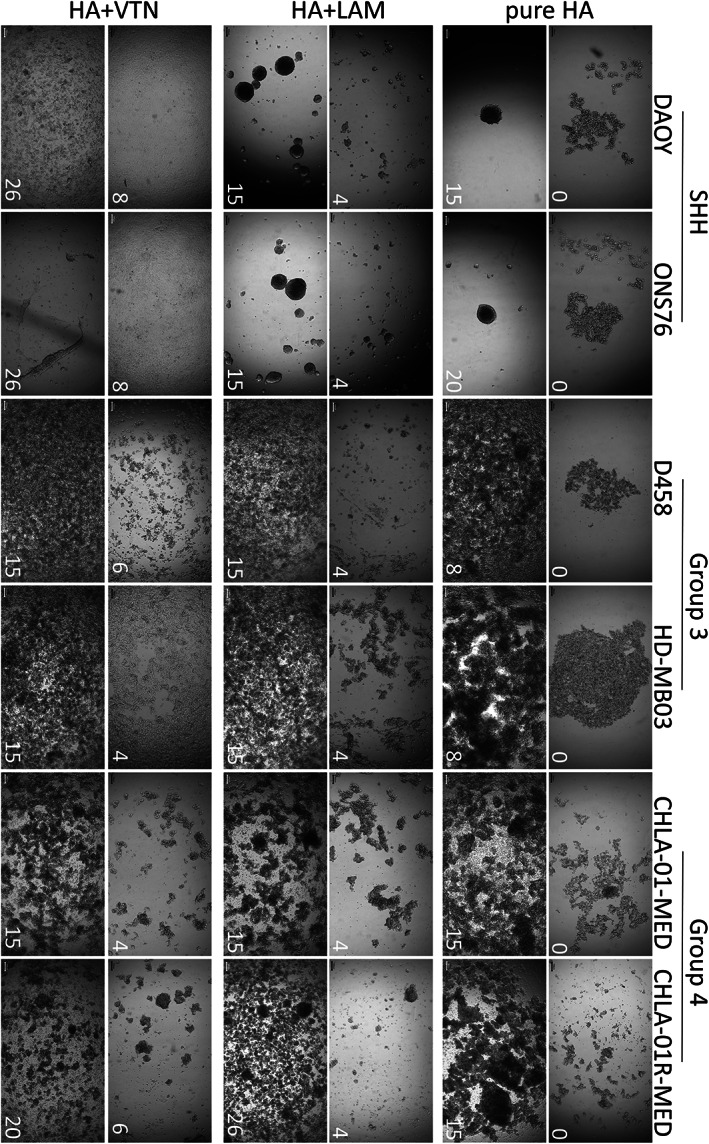
Only Group 3 and Group 4 MB cell lines show laminar growth patterns on HA gels, while SHH cell lines are characterized by nodular growth that can only be altered by vitronectin. MB cell lines representing the different subgroups were seeded on top of pure HA hydrogels or on hydrogels supplemented with either laminin or vitronectin. Growth patterns were monitored over time (number in the bottom right corner equals day of growth). All Group 3 and Group 4 cell lines grow as relatively even laminar monolayers on all three different hydrogels and can cover the whole area within a few weeks. In contrast, SHH cell lines struggle to adhere on pure HA and form a single central spheroid‐like structure instead over time. On laminin‐supplemented HA hydrogels, these cells adhere and form multiple nodules over time. In contrast, the vitronectin‐supplemented hydrogels provide the only surface where SHH MB cell lines grow as a thin laminar monolayer (scale bar = 100 μm). The complete time course for all cell lines can be found in supplementary material, Figures S8–S10.

In order to test the influence of laminin and vitronectin on the MB metastatic growth phenotype, we created composite hydrogels, by combining laminin or vitronectin with HA hydrogel, and assessed the behaviour of cells when seeded on top of the gels (mimicking the leptomeningeal surface) (Figure 4; supplementary material, Figures S8–S10). The growth pattern of both SHH cell lines differed markedly on the different matrix compositions and was distinct from the behaviour observed for Group 3 and Group 4 cell lines. The SHH cell lines (DAOY, ONS76) formed one loosely attached central spheroid‐like structure when seeded on pure HA gels, while a laminin‐containing HA hydrogel induced the growth of multiple fast‐growing nodules. In contrast, vitronectin‐enriched HA hydrogels completely abolished the nodular growth pattern of SHH cell lines and supported growth as a flat, laminar monolayer (Figure 4, left columns). In comparison to the SHH cell lines, Group 3 (HD‐MB03, D458) and Group 4 (CHLA‐01‐MED, CHLA‐01R‐MED) representative cell lines were able to grow and cover the whole gel area as a monolayer on all three matrix compositions (Figure 4, central and right columns). On pure HA gels, Group 4 cell lines attached across the whole gel area as single cells and clusters, while Group 3 cell lines grew as a central flat monolayer. The laminin‐containing HA hydrogel supported initial adherence of single cells that formed clusters and filled the gaps between each other over time with a laminar coating. In comparison to laminin, vitronectin‐supplemented HA hydrogels initiated the flat monolayer growth of Group 3 cell lines. Importantly, vitronectin had a similar effect to laminin on Group 4 cell lines by supporting growth and adherence of single cells, cell clusters, and loose nodules. Our hydrogel model therefore confirms the different roles of laminin and vitronectin in driving MB growth patterns and highlights MB subgroup‐specific adherence and growth differences.

**Figure 5:**
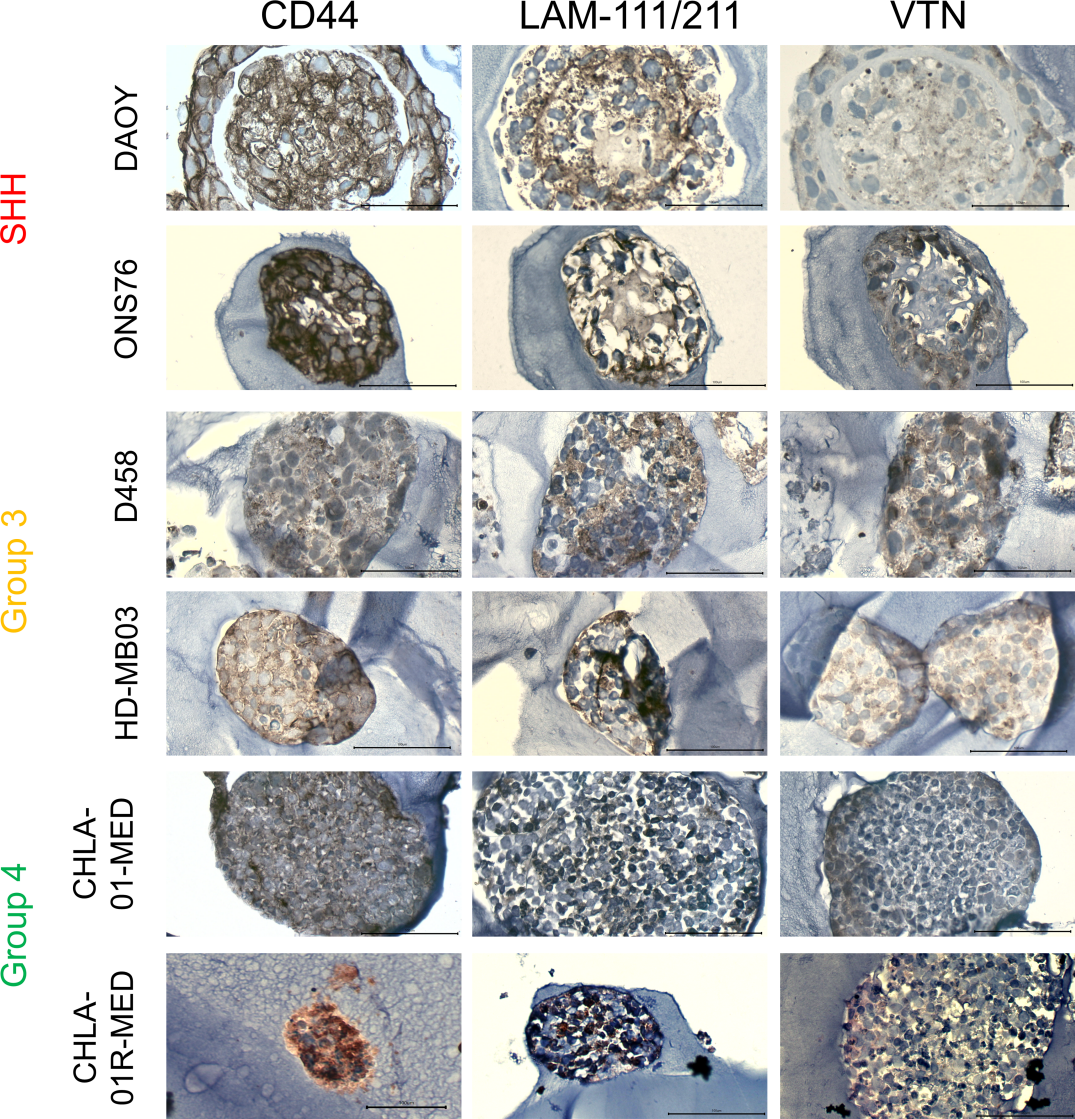
MB tumour nodules grown in pure HA gels express CD44 and secrete the identified ECM components laminin and vitronectin (VTN). MB cell lines representing the different subgroups were grown inside HA hydrogels for 3 weeks, fixed, embedded in paraffin, and stained for CD44, laminin‐111/211, and vitronectin. All cell lines expressed high levels of the HA receptor CD44 (left panel). In contrast, laminin staining was stronger in the relapse than in the primary Group 4 cell line (middle panel). Interestingly, both Group 3 cell lines show diffuse laminin staining, while laminin expression follows a structural pattern in both SHH cell lines. In DAOY and ONS76 nodules, diffuse laminin staining can be found in the core area, while a defined laminin ring separates the central area from the outer ring. Vitronectin staining (right panel) was mainly found at the outer ring of SHH, whereas vitronectin was diffusely expressed throughout Group 3 nodules and the relapse Group 4 nodule (scale bar = 100 μm).

Since we already observed MB subgroup‐specific invasion patterns and chemotherapy responses after long‐term growth in HA hydrogels, we hypothesized that these differences might also reflect a cell line‐specific ECM deposition. To test for the presence of secreted laminin and vitronectin, we processed pure HA hydrogels after 3 weeks of cell encapsulation for immunohistochemistry and stained the nodules for CD44, laminin, and vitronectin (Figure 5). The HA receptor CD44 has been linked to brain tumour progression;[26, 27, 28] hence we chose CD44 as a marker for our MB cell–HA interaction. All cell lines expressed CD44 throughout the whole nodule volume. Although the cell lines had been seeded in pure HA hydrogels without any other ECM factor addition, we found laminin and vitronectin present in the MB cell line nodules. Laminin expression was low in the nodule derived from the primary Group 4 cell line, while it was diffusely expressed in the nodule derived from the Group 4 relapse, as well as in Group 3 cell line‐derived nodules. Interestingly, laminin expression followed a very distinct pattern in SHH nodules. While laminin was diffusely expressed in the central nodule core, it also formed a ring‐like structure that separated the central core from the outer nodule zone, suggesting a structural role of laminin in SHH nodules. Vitronectin was either not present or only secreted at the outer edge of SHH nodules. In contrast, diffuse vitronectin staining was found throughout the whole volume of Group 3 nodules, whilst in Group 4 nodules, expression was initially located at the outer edge of the primary, becoming more diffuse at relapse.

To summarize, ECM factors that have been identified from MB patient's RNA and protein data were expressed and secreted by MB cell lines growing as nodules inside our HA hydrogel model, confirming the biological relevance of our realistic 3D models. Therefore, differences in ECM factor expression level and distribution throughout the nodule might contribute to the observed functional differences in invasion and chemoresistance of the MB subgroups (Figures 1 and 2).

### Identified ECM subtypes predict patient's overall survival and uncover MB subgroup differences

**Figure 6:**
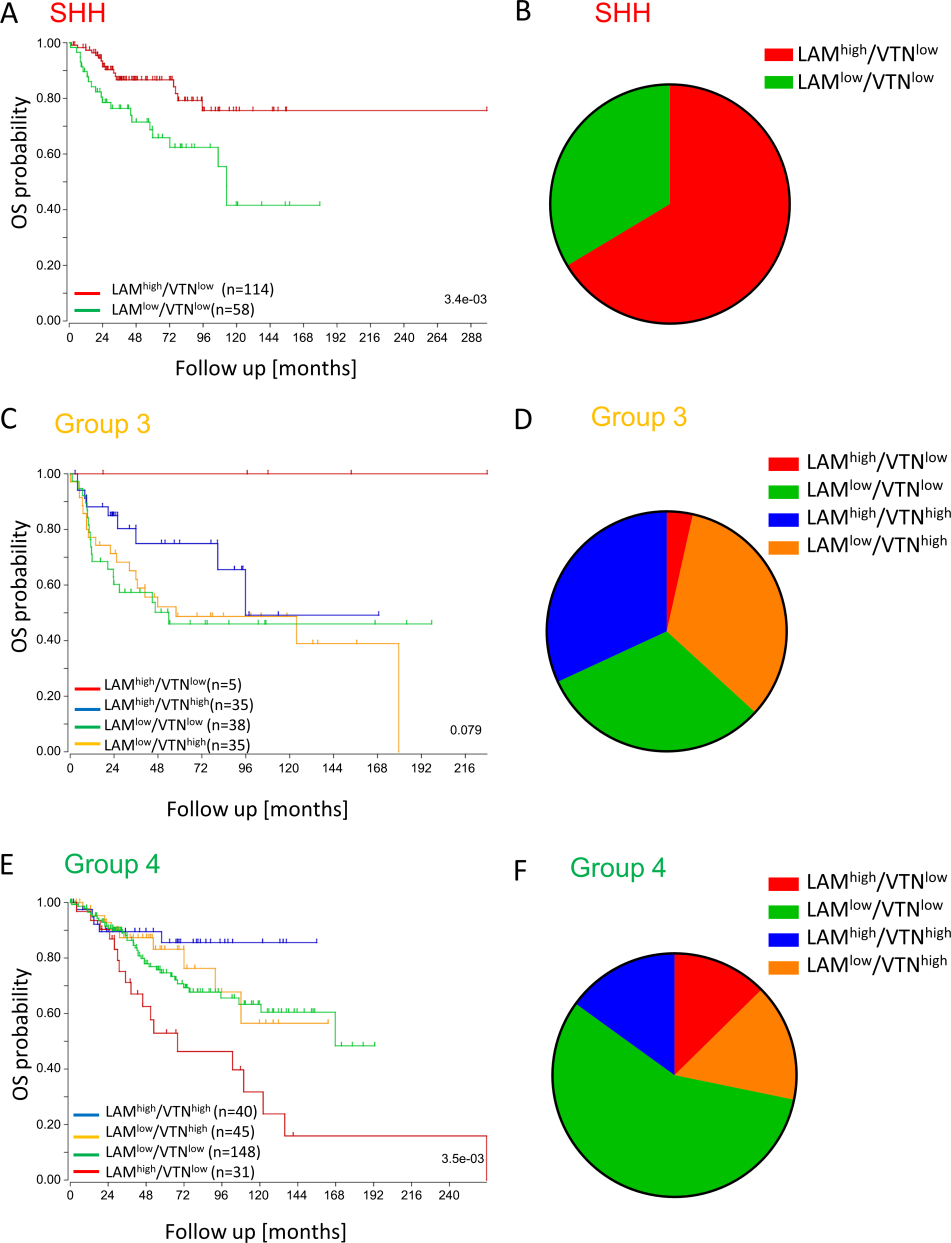
ECM subgroups that are defined by the expression patterns of laminin‐111/211 and vitronectin (VTN) are clinically relevant and predict patient's overall survival (OS) in a subgroup‐specific manner. ECM subgroups have been assigned based on the expression level of *LAMA1/LAMA2* and *VTR* for every SHH, Group 3, and Group 4 patient of the gene expression dataset from Cavalli *et al* [6] (groups with survival data: ‘LAM^high^/VTN^low^’ *n* = 150; ‘LAM^low^/VTN^high^’ *n* = 80; ‘LAM^low^/VTN^low^’ *n* = 244; ‘LAM^high^/VTN^high^’ *n* = 75). (A) In SHH MB, overall survival is good in the laminin group and significantly worse in patients with low expression of *LAMA1/2* and vitronectin (*n* = 172, log‐rank test, *p* = 3.4e−03). (B) The laminin‐dominated ECM subgroup makes up the majority of SHH cases. (C) In Group 3, patients with high vitronectin have the worst overall survival (*n* = 113, log‐rank test, *p* = 0.079). (D) In contrast to SHH, in Group 3 MB the ‘LAM^high^/VTN^low^’ group is very small, while the vitronectin‐containing groups and the LAM^low^/VTN^low^ group account for similar patient's numbers. (E) In Group 4 MB, the worst overall survival is associated with high *LAMA1/2* expression (*n* = 264, log‐rank test, *p* = 3.5e−03). (F) The largest ECM subtype of Group 4 MB shows low expression of both ECM markers.

Having provided evidence that laminin and vitronectin can alter the growth and adhesion patterns of MB subgroups and that both are actively secreted by the tumour cells, survival analysis based on ECM subtypes was then performed using Cavalli *et al*'s data set [6] (Figure 6 and supplementary material, Figure S11). In the SHH subgroup, the ‘LAM^high^/VTN^low^’ subtype correlated with a very good prognosis, while patients with low expression of laminin and vitronectin had a significantly worse survival prognosis (Figure 6A; *p* = 0.003). In contrast, low laminin expression but high levels of vitronectin were associated with the worst overall survival in Group 3 patients (Figure 6B; *p* = 0.079). Interestingly, the five Group 3 patients with high laminin and low vitronectin expression had the best overall survival. While a high laminin and low vitronectin expression defined patients with good overall survival rates in the SHH and Group 3 cohorts, the opposite was true for Group 4, indicating differences in the response to laminin between the MB subgroups (Figure 6C; *p* = 0.004). In Group 4 patients, the ‘LAM^high^/VTN^low^’ subtype correlated with a very poor prognosis, thus identifying a new high‐risk Group 4 subtype. ECM subtypes can therefore be used to identify subgroup‐specific high‐risk patients.

## Discussion

Metastatic dissemination remains the main challenge and reason for therapy failure in MB [1, 29]. Although it is known that MB subgroups differ in metastatic frequency, location, size, and morphology of metastases, we still lack a deep mechanistic understanding of these processes [4, 13, 30]. A favourable brain environment that serves as a hospitable niche is a prerequisite for successful metastatic growth [31, 32]. There is an ongoing discussion regarding whether subpopulations of cancer cells already possess an intrinsic capacity to metastasize or if the neural niche selection pressure causes some cells to evolve and adapt to the new environment [33, 34, 35]. Alternatively, a combination of these events, i.e. bidirectional tumour–brain environment interactions, might explain different morphologies and locations of brain metastases. The presence of MB subgroup‐specific nodular and laminar metastases might therefore reflect subgroup‐dependent biological differences that could present novel targets for therapeutic intervention [13]. Models representing functional MB subgroups would significantly advance our understanding and ability to exploit these differences.

The 3D HA hydrogel model was capable of reproducing clinically relevant MB subgroup invasive phenotypes. Our data suggest that long‐term growth in a realistic matrix allows the cells to alter their environment and set up their specific favourable niche. As a consequence, we observed highly metastatic and chemoresistant behaviour of Group 3 and Group 4 cell lines and pure nodular growth of SHH cell lines. Interestingly, the long‐term drug treatment model with vincristine as well as etoposide revealed resistant cell populations. Those cell populations remained viable post‐treatment and could therefore drive relapse. Our 3D hydrogel model is therefore perfectly suited to studying long‐term drug response and potential resistance mechanisms, for example by comparing cells before and after treatment. Long‐term hydrogel drug screening could therefore become a valuable option to test efficiency and potential resistance mechanisms of drug candidates that have been identified using more high‐throughput assays such as spheroids or neurosphere cultures. Using the model also provides the opportunity to identify and target novel factors involved in MB microenvironmental remodelling.

The glycoproteins laminin and vitronectin are expressed by the cell lines in the HA hydrogel model, correlating with patient's samples at the RNA and protein level, supporting a potential role of laminin and vitronectin in MB metastasis.

The expression and presence of laminin and vitronectin not only predetermine the nodular or laminar growth pattern but also define ECM subtypes. Based on these ECM subtypes, laminin‐expressing, high‐risk Group 4 patients and vitronectin‐expressing, high‐risk Group 3 patients can be identified and important differences between the MB subgroups are highlighted. Interestingly, the high expression of laminin‐111/211 is associated with very good overall survival in SHH and a small proportion of Group 3 patients but with very poor survival in Group 4. This indicates that laminin can have opposing roles depending on the particular cellular context. In cancer and stem cell research, laminins have been found to function as stem cell factors or inducers of differentiation [36, 37, 38, 39, 40, 41]. A better understanding of the cell context‐dependent functions of laminin and its underlying signalling mechanisms will therefore be key for future cancer studies.

For MB, we show that laminin supports the nodular growth of SHH tumours and is a structural component of SHH nodules. It also induces nodule‐like cluster formation of Group 4 cell lines but is not sufficient to alter the Group 3 laminar growth morphology. We hypothesize that SHH and Group 4 tumours share some signalling similarities in response to laminin that eventually induce the nodular phenotype. However, further investigations will be required to understand the obvious differences in laminin‐induced cell signalling between SHH and Group 4 tumours in order to identify novel targets for the identified very high‐risk Group 4 subtype.

In contrast to laminin, we identified vitronectin as an inducer of MB laminar growth. Vitronectin is a serum protein with important functions in inflammation, tissue repair, and homeostatic processes [42]. In glioma, vitronectin is present not only in the serum but also in the cerebrospinal fluid and induces glioma cell migration *in vitro* [42]. Serum levels of vitronectin correlate with glioma grade and predict outcome [43]. In our experiments, vitronectin was only diffusely expressed in Group 3 nodules and correlated with poor overall survival in Group 3 patients. Interestingly, vitronectin gene and protein expression was low in SHH patients but addition of vitronectin to the HA hydrogel induced laminar growth of SHH cell lines. This suggests that responsiveness to ECM factors is similar between MB subgroups but their intrinsic potential to secrete certain ECM factors and therefore remodel their environment differs. Future studies could test if the addition of single ECM components also changes medulloblastoma metastasis in an *in vivo* model using knockout cell lines for the respective ECM factor.

Our study highlights the potential of this research area. The ability to model MB subgroup‐dependent differences in ECM interactions will not only enable us to add a novel functional angle to the existing classification schemes but will also increase our understanding of the fundamental biological differences between MB subgroups and the potential for therapeutic intervention. While conventional 2D cell culture only reveals phenotypical differences between the MB subgroups, 3D hydrogel models also expose functional differences.

## Author contributions statement

FL performed most of the experiments, with MA contributing to specific experiments. AL analysed the RNAseq data. AMG, SST, IDK, MA and CLRM were in involved in research design, manuscript writing, and the final approval. FL and BC designed the research, analysed and interpreted data, and wrote the manuscript.

## Supporting information


**Supplementary materials and methods**
**Figure S1.** Global gene expression of medulloblastoma cell lines correlates with clinical subgroup data
**Figure S2.** Comparison of MB cell line growth in 2D compared with long‐term 3D culture
**Figure S3.** Etoposide (ETO) treatment kills the SHH MB cell line ONS76 effectively, while Group 3 nodules display strong resistance comparable to clinical observations
**Figure S4.** Differential gene expression analysis of the data set from Cavalli *et al* [6] reveals SHH and Group 3 specific ECM markers
**Figure S5.** MB patients express subgroup‐specific levels of laminin (*LAM*) and vitronectin (*VTN*) genes
**Figure S6.** Protein expression of laminin and vitronectin in TMAs
**Figure S7.** Analysis of the data set from Cavalli *et al* [6] reveals subgroup‐specific differences in ECM protein expression patterns
**Figure S8.** Cell growth on pure HA hydrogels reveals remarkable differences between SHH cell lines and Group 3 and Group 4 cell lines
**Figure S9.** Cell growth on laminin‐supplemented HA hydrogels revealed remarkable differences between SHH cell lines and Group 3 and Group 4 cell lines
**Figure S10.** Cell growth on vitronectin‐supplemented HA hydrogels revealed remarkable differences between SHH cell lines and Group 3 and Group 4 cell lines
**Figure S11.** ECM subtypes are composed of different MB subgroup proportions
**Table S1.** Clinicopathological characteristics of MB patients included in the TMAs

## Data Availability

All data generated or analysed during this study are included within the article. The RNA sequencing data have been deposited in the ArrayExpress database at EMBL‐EBI under Annotare accession number E‐MTAB‐9823 (http://www.ebi.ac.uk/arrayexpress).

## References

[1] Taylor MD , Northcott PA , Korshunov A , *et al*. Molecular subgroups of medulloblastoma: the current consensus. Acta Neuropathol 2012; 123 **:** 465–472. 22134537 10.1007/s00401-011-0922-z PMC3306779

[2] Northcott PA , Korshunov A , Witt H , *et al*. Medulloblastoma comprises four distinct molecular variants. J Clin Oncol 2011; 29 **:** 1408–1414. 20823417 10.1200/JCO.2009.27.4324 PMC4874239

[3] Gibson P , Tong Y , Robinson G , *et al*. Subtypes of medulloblastoma have distinct developmental origins. Nature 2010; 468 **:** 1095–1099. 21150899 10.1038/nature09587 PMC3059767

[4] Kool M , Korshunov A , Remke M , *et al*. Molecular subgroups of medulloblastoma: an international meta‐analysis of transcriptome, genetic aberrations, and clinical data of WNT, SHH, Group 3, and Group 4 medulloblastomas. Acta Neuropathol 2012; 123 **:** 473–484. 22358457 10.1007/s00401-012-0958-8 PMC3306778

[5] Sharma T , Schwalbe EC , Williamson D , *et al*. Second‐generation molecular subgrouping of medulloblastoma: an international meta‐analysis of Group 3 and Group 4 subtypes. Acta Neuropathol 2019; 138 **:** 309–326. 31076851 10.1007/s00401-019-02020-0 PMC6660496

[6] Cavalli FMG , Remke M , Rampasek L , *et al*. Intertumoral heterogeneity within medulloblastoma subgroups. Cancer Cell 2017; 31 **:** 737–754.e6. 28609654 10.1016/j.ccell.2017.05.005 PMC6163053

[7] Ramaswamy V , Remke M , Bouffet E , *et al*. Risk stratification of childhood medulloblastoma in the molecular era: the current consensus. Acta Neuropathol 2016; 131 **:** 821–831. 27040285 10.1007/s00401-016-1569-6 PMC4867119

[8] Ellison DW , Kocak M , Dalton J , *et al*. Definition of disease‐risk stratification groups in childhood medulloblastoma using combined clinical, pathologic, and molecular variables. J Clin Oncol 2011; 29 **:** 1400–1407. 20921458 10.1200/JCO.2010.30.2810 PMC3525837

[9] von Hoff K , Hinkes B , Gerber NU , *et al*. Long‐term outcome and clinical prognostic factors in children with medulloblastoma treated in the prospective randomised multicentre trial HIT'91. Eur J Cancer 2009; 45 **:** 1209–1217. 19250820 10.1016/j.ejca.2009.01.015

[10] Zeltzer PM , Boyett JM , Finlay JL , *et al*. Metastasis stage, adjuvant treatment, and residual tumor are prognostic factors for medulloblastoma in children: conclusions from the Children's Cancer Group 921 randomized phase III study. J Clin Oncol 1999; 17 **:** 832–845. 10071274 10.1200/JCO.1999.17.3.832

[11] Holgado BL , Guerreiro Stucklin A , Garzia L , *et al*. Tailoring medulloblastoma treatment through genomics: making a change, one subgroup at a time. Annu Rev Genomics Hum Genet 2017; 18 **:** 143–166. 28475368 10.1146/annurev-genom-091416-035434

[12] Dufour C , Beaugrand A , Pizer B , *et al*. Metastatic medulloblastoma in childhood: Chang's classification revisited. Int J Surg Oncol 2012; 2012 **:** 245385. 22312539 10.1155/2012/245385 PMC3265270

[13] Zapotocky M , Mata‐Mbemba D , Sumerauer D , *et al*. Differential patterns of metastatic dissemination across medulloblastoma subgroups. J Neurosurg Pediatr 2018; 21 **:** 145–152. 29219788 10.3171/2017.8.PEDS17264

[14] Coluccia D , Figuereido C , Isik S , *et al*. Medulloblastoma: tumor biology and relevance to treatment and prognosis paradigm. Curr Neurol Neurosci Rep 2016; 16 **:** 43. 27021772 10.1007/s11910-016-0644-7

[15] Zimmermann DR , Dours‐Zimmermann MT . Extracellular matrix of the central nervous system: from neglect to challenge. Histochem Cell Biol 2008; 130 **:** 635–653. 18696101 10.1007/s00418-008-0485-9

[16] Dauth S , Grevesse T , Pantazopoulos H , *et al*. Extracellular matrix protein expression is brain region dependent. J Comp Neurol 2016; 524 **:** 1309–1336. 26780384 10.1002/cne.23965 PMC7714387

[17] Rauch U . Brain matrix: structure, turnover and necessity. Biochem Soc Trans 2007; 35 **:** 656–660. 17635114 10.1042/BST0350656

[18] Novak U , Kaye AH . Extracellular matrix and the brain: components and function. J Clin Neurosci 2000; 7 **:** 280–290. 10938601 10.1054/jocn.1999.0212

[19] Kim Y , Kumar S . CD44‐mediated adhesion to hyaluronic acid contributes to mechanosensing and invasive motility. Mol Cancer Res 2014; 12 **:** 1416–1429. 24962319 10.1158/1541-7786.MCR-13-0629 PMC4201971

[20] Quail DF , Joyce JA . The microenvironmental landscape of brain tumors. Cancer Cell 2017; 31 **:** 326–341. 28292436 10.1016/j.ccell.2017.02.009 PMC5424263

[21] Bewick V , Cheek L , Ball J . Statistics review 12: survival analysis. Crit Care 2004; 8 **:** 389–394. 15469602 10.1186/cc2955 PMC1065034

[22] Packer RJ , Gajjar A , Vezina G , *et al*. Phase III study of craniospinal radiation therapy followed by adjuvant chemotherapy for newly diagnosed average‐risk medulloblastoma. J Clin Oncol 2006; 24 **:** 4202–4208. 16943538 10.1200/JCO.2006.06.4980

[23] Zhang ZY , Xu J , Ren Y , *et al*. Medulloblastoma in China: clinicopathologic analyses of SHH, WNT, and non‐SHH/WNT molecular subgroups reveal different therapeutic responses to adjuvant chemotherapy. PLoS One 2014; 9 **:** e99490. 24932704 10.1371/journal.pone.0099490 PMC4059646

[24] Yousif LF , Di Russo J , Sorokin L . Laminin isoforms in endothelial and perivascular basement membranes. Cell Adh Migr 2013; 7 **:** 101–110. 23263631 10.4161/cam.22680 PMC3544773

[25] Cote AJ , McLeod CM , Farrell MJ , *et al*. Single‐cell differences in matrix gene expression do not predict matrix deposition. Nat Commun 2016; 7 **:** 10865. 26936319 10.1038/ncomms10865 PMC4782061

[26] Shu C , Wang Q , Yan X , *et al*. Prognostic and microRNA profile analysis for CD44 positive expression pediatric posterior fossa ependymoma. Clin Transl Oncol 2018; 20 **:** 1439–1447. 29704232 10.1007/s12094-018-1876-6

[27] Nishikawa M , Inoue A , Ohnishi T , *et al*. Significance of glioma stem‐like cells in the tumor periphery that express high levels of CD44 in tumor invasion, early progression, and poor prognosis in glioblastoma. Stem Cells Int 2018; 2018 **:** 5387041. 30210550 10.1155/2018/5387041 PMC6126065

[28] Brown DV , Filiz G , Daniel PM , *et al*. Expression of CD133 and CD44 in glioblastoma stem cells correlates with cell proliferation, phenotype stability and intratumor heterogeneity. PLoS One 2017; 12 **:** e0172791. 28241049 10.1371/journal.pone.0172791 PMC5328356

[29] Fernandez‐Teijeiro A , Betensky RA , Sturla LM , *et al*. Combining gene expression profiles and clinical parameters for risk stratification in medulloblastomas. J Clin Oncol 2004; 22 **:** 994–998. 14970184 10.1200/JCO.2004.03.036

[30] Northcott PA , Shih DJH , Peacock J , *et al*. Subgroup‐specific structural variation across 1,000 medulloblastoma genomes. Nature 2012; 488 **:** 49–56. 22832581 10.1038/nature11327 PMC3683624

[31] Calabrese C , Poppleton H , Kocak M , *et al*. A perivascular niche for brain tumor stem cells. Cancer Cell 2007; 11 **:** 69–82. 17222791 10.1016/j.ccr.2006.11.020

[32] Carbonell WS , Ansorga O , Sibson N , *et al*. The vascular basement membrane as ‘soil’ in brain metastasis. PLoS One 2009; 4 **:** e5857. 19516901 10.1371/journal.pone.0005857 PMC2689678

[33] Fidler IJ , Kripke ML . Metastasis results from preexisting variant cells within a malignant tumor. Science 1977; 197 **:** 893–895. 887927 10.1126/science.887927

[34] Talmadge J , Wolman , Fidler I . Evidence for the clonal origin of spontaneous metastases. Science 1982; 217 **:** 361–363. 6953592 10.1126/science.6953592

[35] Hoshide R , Jandial R . The role of the neural niche in brain metastasis. Clin Exp Metastasis 2017; 34 **:** 369–376. 28905151 10.1007/s10585-017-9857-7

[36] Lathia JD , Li M , Hall PE , *et al*. Laminin alpha 2 enables glioblastoma stem cell growth. Ann Neurol 2012; 72 **:** 766–778. 23280793 10.1002/ana.23674 PMC3615417

[37] De Arcangelis A , Lefebvre O , Méchine‐Neuville A , *et al*. Overexpression of laminin α1 chain in colonic cancer cells induces an increase in tumor growth. Int J Cancer 2001; 94 **:** 44–53. 11668477 10.1002/ijc.1444

[38] Qin Y , Rodin S , Simonson OE , *et al*. Laminins and cancer stem cells: partners in crime? Semin Cancer Biol 2017; 45 **:** 3–12. 27491691 10.1016/j.semcancer.2016.07.004

[39] Horejs C‐M , Serio A , Purvis A , *et al*. Biologically‐active laminin‐111 fragment that modulates the epithelial‐to‐mesenchymal transition in embryonic stem cells. Proc Natl Acad Sci U S A 2014; 111 **:** 5908–5913. 24706882 10.1073/pnas.1403139111 PMC4000797

[40] Jin L , Feng T , Shih HP , *et al*. Colony‐forming cells in the adult mouse pancreas are expandable in Matrigel and form endocrine/acinar colonies in laminin hydrogel. Proc Natl Acad Sci U S A 2013; 110 **:** 3907–3912. 23431132 10.1073/pnas.1301889110 PMC3593860

[41] Yao Y , Chen Z‐L , Norris EH , *et al*. Astrocytic laminin regulates pericyte differentiation and maintains blood brain barrier integrity. Nat Commun 2014; 5 **:** 3413. 24583950 10.1038/ncomms4413 PMC3992931

[42] Leavesley DI , Kashyap AS , Croll T , *et al*. Vitronectin – master controller or micromanager? IUBMB Life 2013; 65 **:** 807–818. 24030926 10.1002/iub.1203

[43] Chen M‐H , Lu C , Sun J , *et al*. Diagnostic and prognostic value of serum vitronectin levels in human glioma. J Neurol Sci 2016; 371 **:** 54–59. 27871448 10.1016/j.jns.2016.10.022

[44] Hynes WF , Doty NJ , Zarembinski TI , *et al*. Micropatterning of 3D microenvironments for living biosensor applications. Biosensors 2014; 4 **:** 28–44. 24791214 10.3390/bios4010028 PMC4004032

[45] Vanderhooft JL , Alcoutlabi M , Magda JJ , *et al*. Rheological properties of cross‐linked hyaluronan‐gelatin hydrogels for tissue engineering. Macromol Biosci 2009; 9 **:** 20–28. 18839402 10.1002/mabi.200800141 PMC2711643

[46] Andrews S. FastQC: A Quality Control Tool for High Throughput Sequence Data. [Accessed 7 October 2020]. Available from: http://www.bioinformatics.babraham.ac.uk/projects/fastqc/

[47] Martin M . Cutadapt removes adapter sequences from high‐throughput sequencing reads. EMBnet J 2011; 17 **:** 10–12.

[48] Dobin A , Davis CA , Schlesinger F , *et al*. STAR: ultrafast universal RNA‐seq aligner. Bioinformatics 2013; 29 **:** 15–21. 23104886 10.1093/bioinformatics/bts635 PMC3530905

[49] Liao Y , Smyth GK , Shi W . featureCounts: an efficient general purpose program for assigning sequence reads to genomic features. Bioinformatics 2014; 30 **:** 923–930. 24227677 10.1093/bioinformatics/btt656

[50] Robinson MD , McCarthy DJ , Smyth GK . edgeR: a Bioconductor package for differential expression analysis of digital gene expression data. Bioinformatics 2010; 26 **:** 139–140. 19910308 10.1093/bioinformatics/btp616 PMC2796818

[51] Affymetrix . Analysis Power Tools (APT). [Accessed 13 October 2020]. Available from: https://www.affymetrix.com/support/developer/powertools/changelog/index.html

[52] Thompson JA , Tan J , Greene CS . Cross‐platform normalization of microarray and RNA‐seq data for machine learning applications. PeerJ 2016; 4 **:** e1621. 26844019 10.7717/peerj.1621 PMC4736986

[53] Johnson WE , Li C , Rabinovic A . Adjusting batch effects in microarray expression data using empirical Bayes methods. Biostatistics 2007; 8 **:** 118–127. 16632515 10.1093/biostatistics/kxj037

[54] Pinto MP , Jacobsen BM , Horwitz KB . An immunohistochemical method to study breast cancer cell subpopulations and their growth regulation by hormones in three‐dimensional cultures. Front Endocrinol (Lausanne) 2011; 2 **:** 15. 22649363 10.3389/fendo.2011.00015 PMC3355989

