# Supplementary material for: 3D hydrogels reveal medulloblastoma subgroup differences and identify extracellular matrix subtypes that predict patient outcome
Source: J Pathol. 2020 Dec 17;253(3):326–38. doi: 10.1002/path.5591 (PMC7986745; doi:10.1002/path.5591)
Supplement: Supplementary file 1 — Supplementary materials and methods Figure S1. Global gene expression of medulloblastoma cell lines correlates with clinical subgroup data Figure S2. Comparison of MB cell line growth in 2D compared with long‐term 3D culture Figure S3. Etoposide (ETO) treatment kills the SHH MB cell line ONS76 effectively, while Group 3 nodules display strong resistance comparable to clinical observations Figure S4. Differential gene expression analysis of the data set from Cavalli et al [6] reveals SHH and Group 3 specific ECM markers Figure S5. MB patients express subgroup‐specific levels of laminin (LAM) and vitronectin (VTN) genes Figure S6. Protein expression of laminin and vitronectin in TMAs Figure S7. Analysis of the data set from Cavalli et al [6] reveals subgroup‐specific differences in ECM protein expression patterns Figure S8. Cell growth on pure HA hydrogels reveals remarkable differences between SHH cell lines and Group 3 and Group 4 cell lines Figure S9. Cell growth on laminin‐supplemented HA hydrogels revealed remarkable differences between SHH cell lines and Group 3 and Group 4 cell lines Figure S10. Cell growth on vitronectin‐supplemented HA hydrogels revealed remarkable differences between SHH cell lines and Group 3 and Group 4 cell lines Figure S11. ECM subtypes are composed of different MB subgroup proportions Table S1. Clinicopathological characteristics of MB patients included in the TMAs [file PATH-253-326-s001.docx]

**3D hydrogels reveal medulloblastoma subgroup differences and identify extracellular matrix subtypes that predict patient outcome**

F Linke *et al. J Pathol* DOI: 10.1002/path.5591

**Supplementary materials and methods**

**Supplementary Figures S1–S11**

**Supplementary Table S1**

**Supplementary materials and methods**

Reference numbers refer to the main text list

*Medulloblastoma cell lines, culturing, and reagents*

The DAOY cell line was obtained from the ATCC (Manassas, VA, USA), ONS76 from Annette Künkele (Charité Universitätsmedizin Berlin, Germany), HD-MB03 from Dr Till Milde (DKFZ Heidelberg, Germany), D458 from John R Silber (University of Washington, Seattle, WA, USA), and CHLA-01-MED and CHLA-01R-MED from Professor Geoff Pilkington (University of Portsmouth, Portsmouth, UK). DAOY and D458 were grown in DMEM (Thermo Fisher, Waltham, MA, USA) with 10% FBS (HyClone, Thermo Fisher); ONS76 and HD-MB03 were grown in RPMI 1640 (Sigma-Aldrich, St Louis, MO, USA) with 10% FBS; and CHLA-01-MED and CHLA-01R-MED were grown in DMEM/F12 supplemented with B27, 20 ng/ml EGF, and 20 ng/ml bFGF (all Thermo Fisher). All cells were maintained at 37 °C in a humidified atmosphere containing 5% CO_2_. During the course of this study, all cell lines were routinely confirmed to be mycoplasma-negative (MycoAlert, Lonza, Basel, Switzerland).

*HA hydrogel preparation, long-term cell culture, and quantification of invasion*

Hyaluronan (1%) and Extralink (2% PEGDA) concentrations were chosen in order to achieve a matrix stiffness around 1.5 kPa and free diffusion of particles less than 75 kDa [44,45]. For long-term embedding experiments, an approximately 1-mm-thick pure gel layer (30 µl) was pipetted and equally distributed in each well of a 96-well plate. After the bottom gel layer had set, a central cell-containing gel layer (40 µl) was added (DAOY, ONS76, D458, HD-MB03: 10 000 cells per gel; CHLA-01-MED, CHLA-01R-MED: 50 000 cells per gel; Group 4 cell lines grow as suspensions consisting of cell clusters and single cells). The ‘gel sandwich’ was completed by adding a pure gel layer on top (30 µl). Each gel layer was allowed to set properly before the next layer was added. Finally, each ‘gel sandwich’ was covered with 100 µl of the appropriate medium and cells were allowed to grow over weeks. The medium was replaced every 2 or 3 days. All gels were monitored daily and images were captured.

An invasion event was defined if growing cells were detected either on the well bottom or growing on the gel surface. The occurrence of these invasion events as well as the time point was monitored for each cell line from at least 20 gels of at least three independent gel preparations.

In order to analyse the laminin or vitronectin-induced changes in adhesion and growth patterns, cells were seeded on top of laminin- or vitronectin-containing hydrogels. Hystem gels were prepared with the additional incorporation of 16.7 µg/ml laminin I or vitronectin (both Cultrex). After the gels had set (50 µl per 96-well), the cell suspension was added and the plate was not moved for the following 16 h to allow cell attachment. All gels were monitored daily, imaged, and the experiment was ended once the cells had covered the entire gel area.

*Etoposide drug treatment in 3D*

To confirm a different drug response between the SHH and Group 3 hydrogel model, etoposide (S1225; Selleckchem, Munich, Germany; 588 Da) was added to the cell culture medium of 3-week-old hydrogels at three different concentrations in DMSO. The etoposide concentrations chosen were the IC_50_ of a single VCR dosage (0.5 µm) as well as the two-fold (1 µm) and ten-fold IC_50_ (5 µm) as defined in spheroid cultures. Vehicle control (DMSO) was also used. Etoposide renewal was performed 24, 72, and 144 h after the first dosage. In order to assess the cell’s recovery potential, all gels were washed and covered with fresh, drug-free medium 1 week after the final etoposide treatment and monitored for a further 4 weeks.

*Western blotting*

Cell pellets (at least 100 000 cells) were lysed in NP-40 lysis buffer [50 mM Tris (pH 7.4), 150 mm NaCl, 1 mm EDTA, 0.5% NP-40], and 20 µg of total protein was used for SDS PAGE. After SDS PAGE size-fractionation of proteins, they were transferred to Immobilon PVDF membranes (Millipore, Burlington, MA, USA). Membranes were incubated in blocking buffer (5% milk powder, 0.1% Tween in Tris-buffered saline) at room temperature for 1 h and afterwards incubated with primary antibody in blocking buffer overnight at 4 °C. After washing, blots were incubated with their respective peroxidase-coupled secondary antibody for 1 h at room temperature. Enhanced chemiluminescence detection was used to visualize the immune reaction (Image Reader LAS-4000; Fujifilm, Minato, Tokyo, Japan). Primary antibodies against C-MYC (D84C12/5605, 1:1000; Cell Signaling Technology, Danvers, MA, USA) and GAPDH (14C10/2118, 1:2000; Cell Signaling Technology) were used. The secondary antibody was goat anti-rabbit IgG (heavy and light chain) antibody conjugated to horseradish peroxidase (7074, 1:2000; Cell Signaling Technology).

*RNA isolation and RNA sequencing*

Cell pellets of at least 100 000 cells were washed with HBSS and frozen in liquid nitrogen. For homogenization, ceramic spheres (Lysing Matrix D; MP Biomedicals, Santa Ana, CA, USA) and the FastPrep-24 homogenizer (MP Biomedicals, speed 4 m/s, tube holder MP:24*2, and time 20 s) were used. Total RNA was isolated from 2D pellets using the NucleoSpin RNA Plus Kit (Macherey-Nagel, Düren, Germany) according to the manufacturer’s instructions. In total, three biological replicates of each cell line were processed respectively. RNA amount was determined using the Qubit RNA BR kit with the Qubit 4 (both Thermo Fisher).

Library preparation and RNA sequencing (transcriptome sequencing including lncRNA on Illumina PE150) were performed by Novogene Company Limited (Cambridge, UK). Samples with less than 100 ng or with non-qualifying RIN values were excluded from the sequencing. All prepared libraries successfully passed Novogene’s internal quality control checks and were sequenced. Following sequencing, quality control of the sequencing data was performed which confirmed that all samples had high quality scores, indicating good technical performance of the sequencing.

*RNA sequence data analysis*

We used FastQC to perform quality checks of raw RNA data, followed by adapter and low quality read filtering using the Cutadapt package (version 1.16.6) [46,47]. The trimmed paired-end sequences were aligned with the human genome (hg38, GenBank assembly accession: GCA_000001405.28) and Gencode annotation (v35) using the STAR (version 2.7.5b) alignment tool [48]. Unique reads from genomic alignment were processed and we used the featureCount tool [49] for transcript abundance quantification. STAR read counts were used as input into edgeR [50]. Genes with read counts greater than 10 in three or more samples were kept for subsequent analyses. After normalization analyses, counts per million (cpm) on a log_2_ scale were used for downstream exploratory analyses.

*Microarray data analysis*

The previously published gene expression profiling of 763 primary medullobalstoma samples was downloaded from the Gene Expression Omnibus (GEO) database (GSE85217). Gene-level (core meta-probeset) expression values were calculated for the 763 Affymetrix Human Gene 1.1 ST array CEL files using Affymetrix Power Tools (APT) [51]. Arrays were normalized using RMA, which included RMA background correction, quantile normalization, log transformation, and probeset summarization. Detection above background (DABG) was performed at both the probe and the probeset level using GC-matched background probes, and low variance probesets were excluded. Normalized expression values on a log_2_ scale were used for subsequent analyses.

*Combined data analysis*

We used the training distribution matching (TDM) method [52] to transform the RNA-seq data for the cross-platform normalization with microarray data followed by ComBat, an empirical Bayes method to correct for batch effects between RNA-seq and microarray datasets [52,53]. To detect robust clustering of cell lines with the medulloblastoma patients, we used principal component analysis (PCA). For the PCA, the combined dataset was reduced to 2000 genes exhibiting the largest median absolute deviation (MAD).

*Immunohistochemistry of HA hydrogels and tissue microarrays*

HA hydrogels were removed from their 96-well plates and embedded in Histogel (Thermo Fisher) as described by Pinto *et al* [54]. The Histogel blocks were incubated on ice for 10 min, fixed in 4 % paraformaldehyde, and embedded in paraffin wax. Sections were then processed for immunohistochemistry. Primary antibodies against CD44 (ab157107, 1:500; Abcam, Cambridge, UK), laminin 1+2 (ab7463, 1:500; Abcam), and vitronectin (ab45139, 1:100; Abcam) were used in combination with a goat anti-rabbit IgG H&L secondary antibody (ab214880; Abcam). For laminin 111/211 staining, pepsin digestion was performed, and for CD44 and vitronectin, heat-induced antigen retrieval.

For MB tissue microarray (TMA) analysis, 42 tumour cores of known MB subgroups were stained (SHH: *n*= 11; Group 3: *n*= 6; Group 4: *n*= 25). Clinical and histological data are presented in supplementary material, Table S1. IHC scoring was performed by two independent assessors (IHC score per core: 0: no staining; 1: <10% positive staining; 2: 10–49% positive staining; 3: 50–80% positive staining; 4: >80% positive staining). The TMA studies, and the experimental protocols required, were reviewed and approved by the National Research Ethics Service Committee East Midlands – Nottingham 2 and have therefore been performed in accordance with the ethical standards laid down in an appropriate version of the 1975 Declaration of Helsinki, as revised in 1983 (REC reference 11/EM/0076). For all patients, informed consent was obtained from the patient, or a parent and/or legal guardian where the patient was under 18 years of age, prior to their inclusion in the study.

*ECM protein enrichment analysis, ECM subtype definition, and overall survival analysis*

Analysis of available microarray mRNA expression and survival data was conducted using the R2: Genomics Analysis and Visualization Platform (http://r2.amc.nl). The Cavalli medulloblastoma data set was analysed [6]. In this study, 763 fresh-frozen primary MB samples were analysed using the Affymetrix Human Gene 1.1 ST Array. For ECM protein enrichment analysis, the expression of genes of the KEGG gene set ‘ECM receptor interaction’ (M7098) was compared between SHH and Group 3, SHH and Group 4, and Group 3 and Group 4 (ANOVA with FDR correction; *p* < 0.01). A comparison of the resulting gene lists revealed three genes with highest expression in Group 3 and lowest expression in SHH, and seven genes with the opposite expression pattern (supplementary material, Figure S3).

For ECM subtype assignment, *VTN* and *LAMA1/A2* gene expression was considered for each patient and normalized with the mean of the whole cohort [normalized gene expression A = (gene expression A – mean cohort expression of gene A)/mean cohort expression of gene A]. Four ECM subtypes were assigned based on the resulting algebraic signs of the normalized VTN and LAMA1/A2 expression: LAM^high^/VTN^low^, LAM^low^/VTN^high^, LAM^high^/VTN^high^, and LAM^low^/VTN^low^.

Kaplan–Meier overall survival analysis of the MB patients and comparison between the defined ECM subtypes were performed using the log-rank test as described by Bewick *et al* [21] (R2: Genomics Analysis and Visualization Platform).

**Supplementary Figures S1–S11**


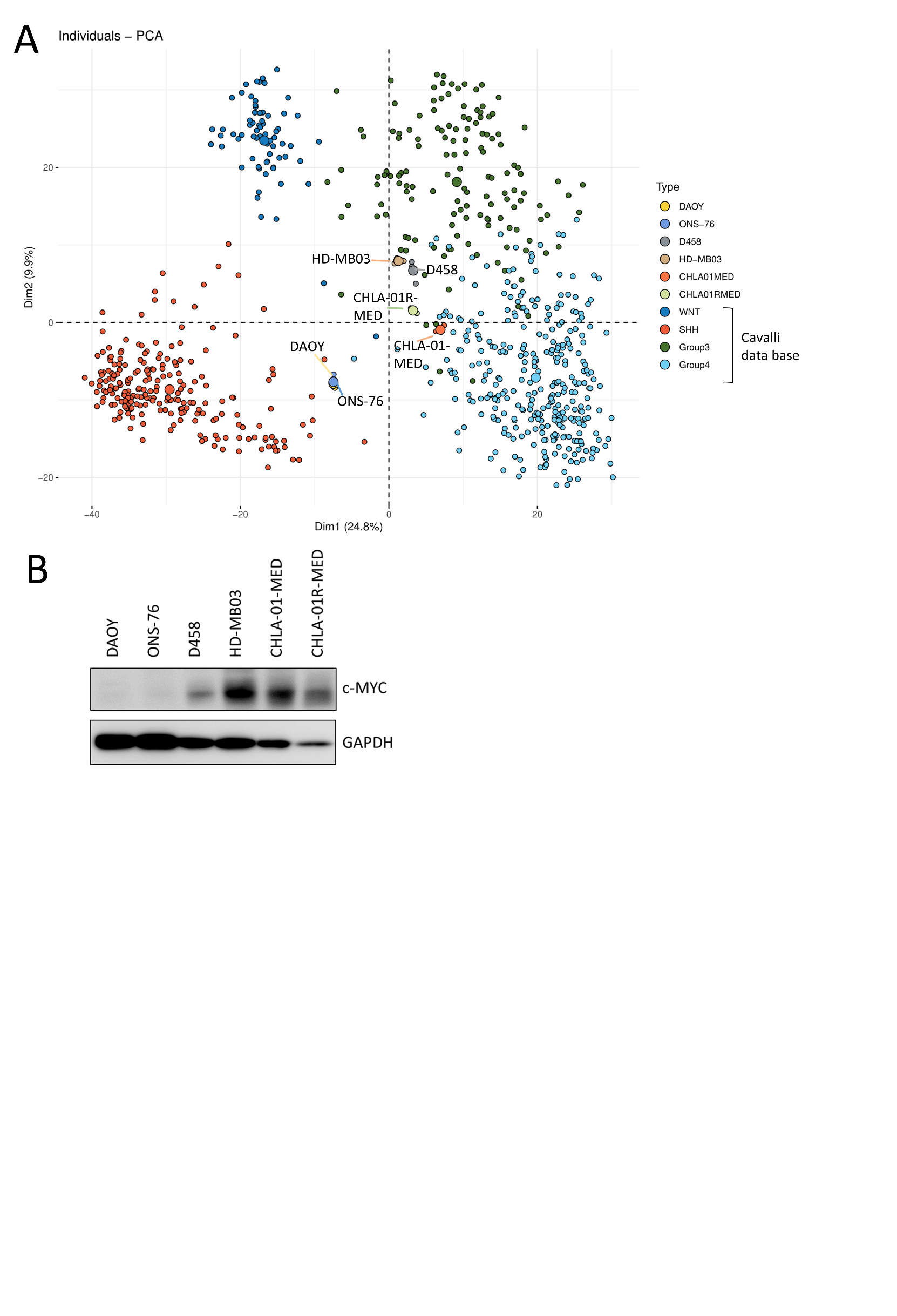


**Figure S1. Global gene expression of medulloblastoma cell lines correlates with clinical subgroup data.** (A) RNA sequencing of the six medulloblastoma cell lines, DAOY, ONS76, D458, HD-MB03, CHLA-01-MED, CLA-01R-MED, was performed and gene expression was correlated with the publicly available data set from Cavalli et al [6]. Principal component analysis (PCA) showed close clustering of both SHH cell lines, DAOY and ONS76, with the SHH patient’s cohort, while HD-MB03 and D458 cells cluster with Group 3 and CHLA-01/01R-Med cluster with Group 4 patients. (B) Western blotting of all six cell lines confirmed that c-MYC (57 kDa) expression was high in Group 3/Group 4 cell lines but very low in both SHH cell lines. GAPDH (37 kDa) was used as a loading control.


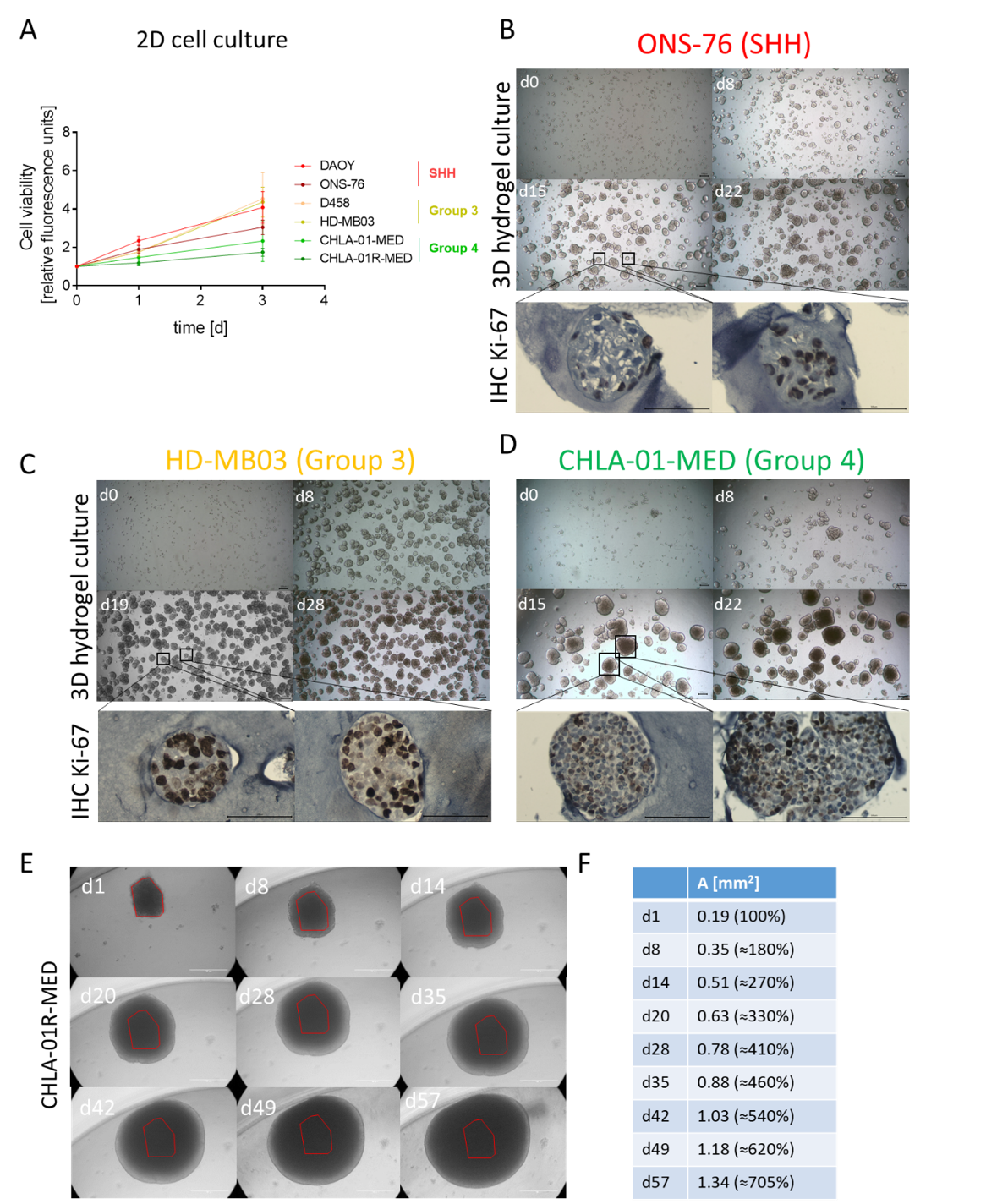


**Figure S2. Comparison of MB cell line growth in 2D compared with long-term 3D culture.** (A) The different MB cell lines grow on plastic flasks in a cell line-specific manner that poorly replicates in vivo tumour-specific behaviour. Cell growth is lowest in both Group 4 cell lines (CHLA-01-MED, CHLA-01R-MED) and highest in Group 3 and one SHH cell line (D458, HD-MB03, DAOY) after 3 days [mean ± SEM, n = 3 (DAOY, ONS76, HD-MB03, CHLA-01-MED, CHLA-01R-MED); n = 2 (D458)]. MB cell lines representative of the subgroups SHH (B; ONS76), Group 3 (C; HD-MB03), and Group 4 (D; CHLA-01-MED) grow to form large nodules over weeks when they are encapsulated in HA hydrogels. IHC staining of nodule sections for the proliferation marker Ki-67 confirmed the presence of actively dividing cells inside the nodules (bottom panel; scale bar = 100 µm). (E) Nodular growth of the Group 4 cell line CHLA-01R-MED was observed over 2 months. The perimeter of the original seeded cell cluster is shown in red (scale bar=500 µm). (F) After 7 weeks, the measurable area exceeded 1 mm^2^.


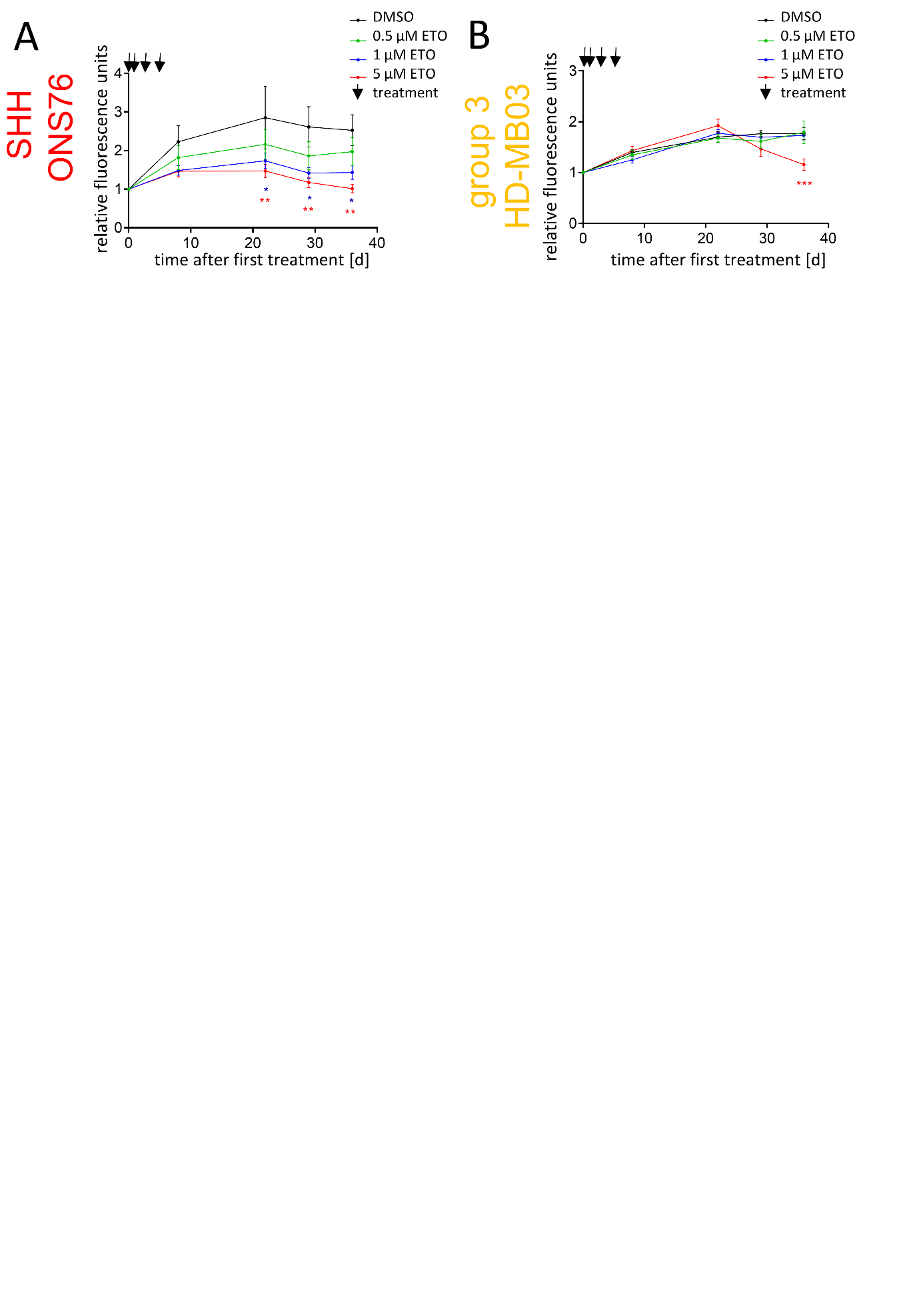


**Figure S3. Etoposide (ETO) treatment kills the SHH MB cell line ONS76 effectively, while Group 3 nodules display strong resistance comparable to clinical observations.** The ETO concentrations chosen were the IC_50_ of a single ETO dosage (0.5 µm) as well as the two-fold (1 µm) and ten-fold IC_50_ (5 µm). After 3 weeks of growth inside the HA hydrogels, the SHH cell line ONS76 (A) and the Group 3 cell line HD-MB03 (B) were treated four times with either 0.5 µm, 1 µm or 5 µm ETO or vehicle during 1 week and cell viability was monitored for the following 4 weeks after removal of drug/vehicle. While 1 µm and 5 µm ETO significantly reduced cell viability in the SHH cell line, only 5 µm ETO significantly affected Group 3 cell viability with a delayed response 2 weeks after the treatment ended (mean ± SEM; n = 3; two-way ANOVA and Dunnett’s post hoc test; *p < 0.05, **p < 0.01, and ***p < 0.001).


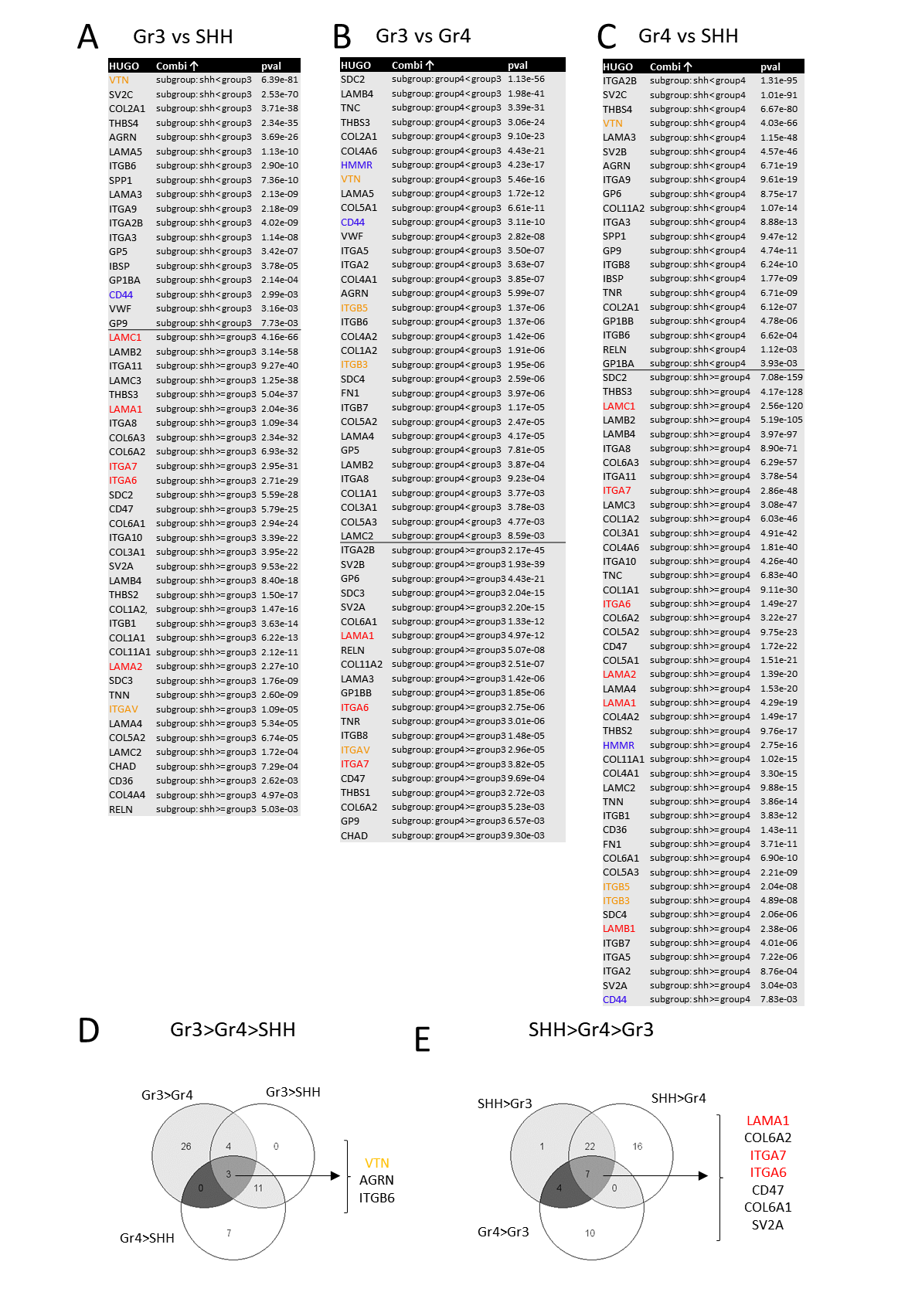


**Figure S4. Differential gene expression analysis of the data set from Cavalli et al [6] reveals SHH and Group 3 specific ECM markers.** The KEGG gene set ‘ECM receptor interaction’ was used to identify genes differentially expressed between (A) Group 3 and SHH, (B) Group 3 and Group 4, and (C) Group 4 and SHH. Genes relevant for HA interaction are shown in blue, for laminin-111/-211 in red, and for vitronectin in orange. ANOVA with FDR correction; p < 0.01. (D) By comparing the resulting gene lists, three genes with highest expression in Group 3, intermediate expression in Group 4, and lowest expression in SHH were identified. (E) The opposite comparison revealed seven target genes that were expressed highest in SHH, intermediate in Group 4, and lowest in Group 3.


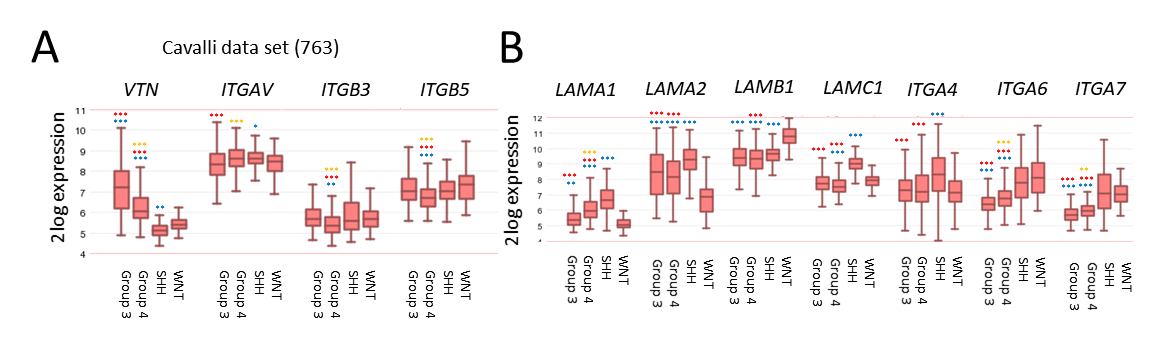


**Figure S5. MB patients express subgroup-specific levels of laminin (LAM) and vitronectin (VTN) genes.** ECM component/receptor gene expression analysis of the data set from Cavalli et al [6] is presented according to the subgroups (WNT: n = 70; SHH: n = 223; Group 3: n = 144; Group 4: n = 326). The KEGG gene set ‘ECM receptor interaction’ was used to identify genes with highest expression in Group 3, intermediate expression in Group 4, and lowest expression in SHH tumours and vice versa (see full gene comparison lists in supplementary material, Figure S3). (A) Vitronectin (VTN) is one of three genes that is explicitly highly-expressed in Group 3 tumours and lowest in SHH (full analysis in supplementary material, Figure S3). Gene expression data for VTN and VTN-binding integrin (ITG) receptor subunits are presented. Note the high ITG gene expression levels of Group 3 and SHH tumours in contrast to Group 4 [Kruskal–Wallis test with Dunn’s post hoc test; *p < 0.05, **p < 0.01, and ***p < 0.001 with significance compared to WNT (blue asterisks), SHH (red asterisks) or Group 3 (yellow asterisks)]. (B) One of seven genes with explicitly high expression in SHH was LAMA1 (full analysis in supplementary material, Figure S3). Gene expression data of laminin-111/laminin-211 and integrin receptors suggest a particular function in SHH MB tumours. The expression of LAMA1, LAMA2, LAMC1, ITGA4, ITGA6, and ITGA7 are specifically high in SHH and low in Group 3 [Kruskal–Wallis test with Dunn’s post hoc test; *p < 0.05, **p < 0.01, and ***p < 0.001 with significance compared to WNT (blue asterisks), SHH (red asterisks) or Group 3 (yellow asterisks)].


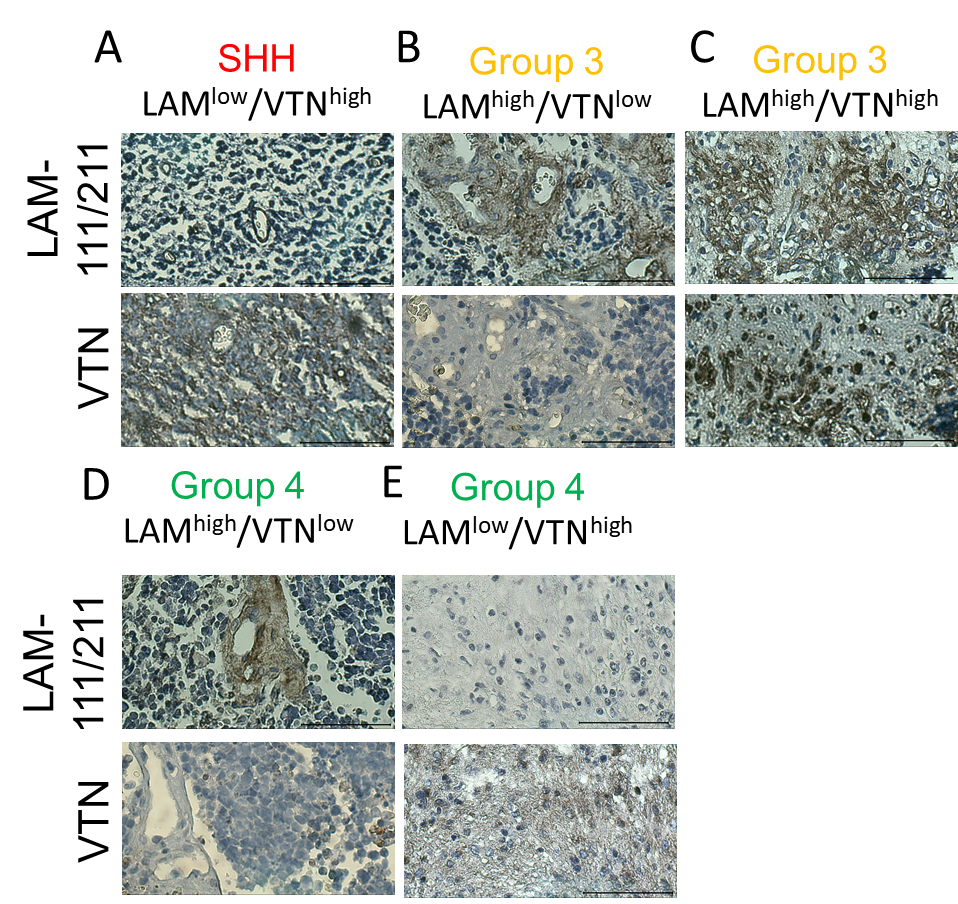


**Figure S6. Protein expression of laminin and vitronectin in TMAs.** (A) An example of a ‘LAM^low^/VTN^high^’ SHH tumour with low laminin expression. (B) An example of a ‘LAM^high^/VTN^low^’ Group 3 MB tumour with only low vitronectin expression. (C) In contrast, another Group 3 tumour shows high laminin expression in addition to high/moderate staining for vitronectin. (D) An example for a ‘LAM^high^/VTN^low^’ Group 4 tumour is shown. (E) Another exemplar of a Group 4 tumour showing low expression of laminin and high expression of vitronectin (scale bar = 100 µm).


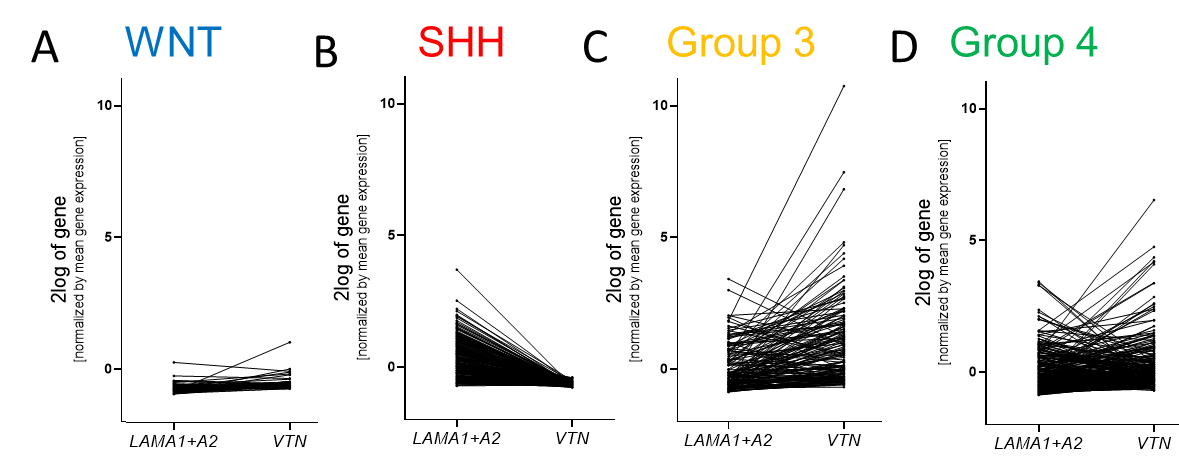


**Figure S7. Analysis of the data set from Cavalli et al [6] reveals subgroup-specific differences in ECM protein expression patterns.** (A) The WNT subgroup is mainly characterized by ‘LAM^low^/VTN^low^’ cases. (B) ‘LAM^high^/VTN^low^’ cases can be found in the SHH subgroup. (C) Group 3 patients show ‘LAM^low^/VTN^high^’, but also equivalent expression patterns of laminin and vitronectin. (D) In Group 4, the predominant patterns are ‘LAM^high^/VTN^low^’ and ‘LAM^low^/VTN^high^’. (WNT: n = 70; SHH: n = 223; Group 3: n = 144; Group 4: n = 326.)


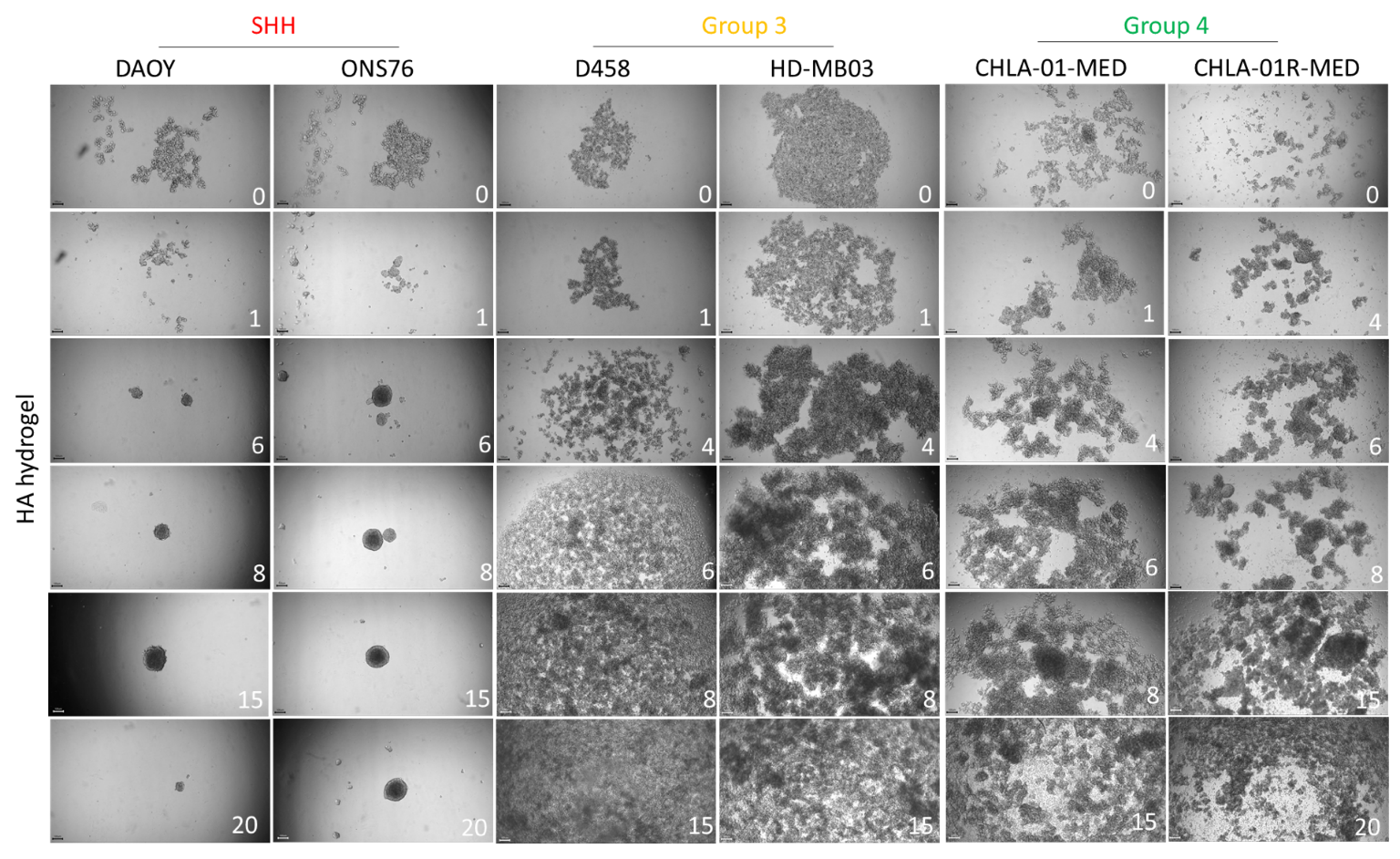


**Figure S8. Cell growth on pure HA hydrogels reveals remarkable differences between SHH cell lines and Group 3 and Group 4 cell lines.** Growth patterns were monitored over time (number in the bottom right corner equals day of growth). SHH cell lines (DAOY, ONS76) formed central spheroid-like structures during the first week and then grew over time. In contrast, Group 3 cell lines (D458, HD-MB03) stayed as flat monolayers and covered the whole gel over time. Group 4 cell lines (CHLA-01-MED, CHLA-01R-MED) behaved similarly to Group 3 cell lines, but started growing in patches and filled up the space inbetween over time (scale bar = 100 µm).


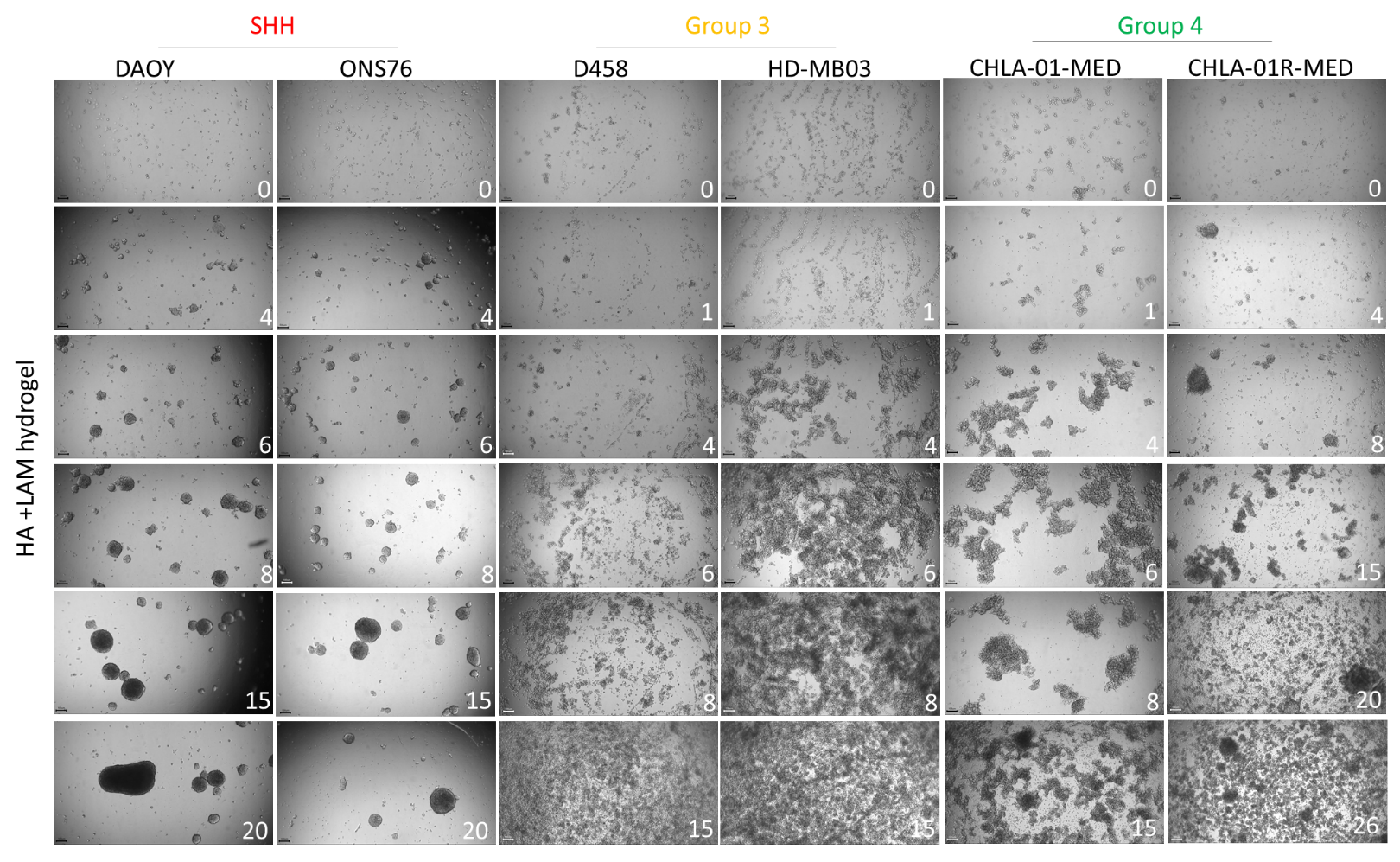


**Figure S9. Cell growth on laminin-supplemented HA hydrogels revealed remarkable differences between SHH cell lines and Group 3 and Group 4 cell lines.** Growth patterns were monitored over time (number in the bottom right corner equals day of growth). SHH cell lines (DAOY, ONS76) formed multiple nodules over the entire gel area. These nodules grew over time or merged. Group 3 cell lines (D458, HD-MB03) adhered as single cells across the entire gel area and filled the gaps between each other over time with a laminar coating. Group 4 cell lines (CHLA-01-MED, CHLA-01R-MED) behaved similarly as on the pure HA hydrogels and started growing in patches and filled up the space inbetween over time (scale bar = 100 µm).


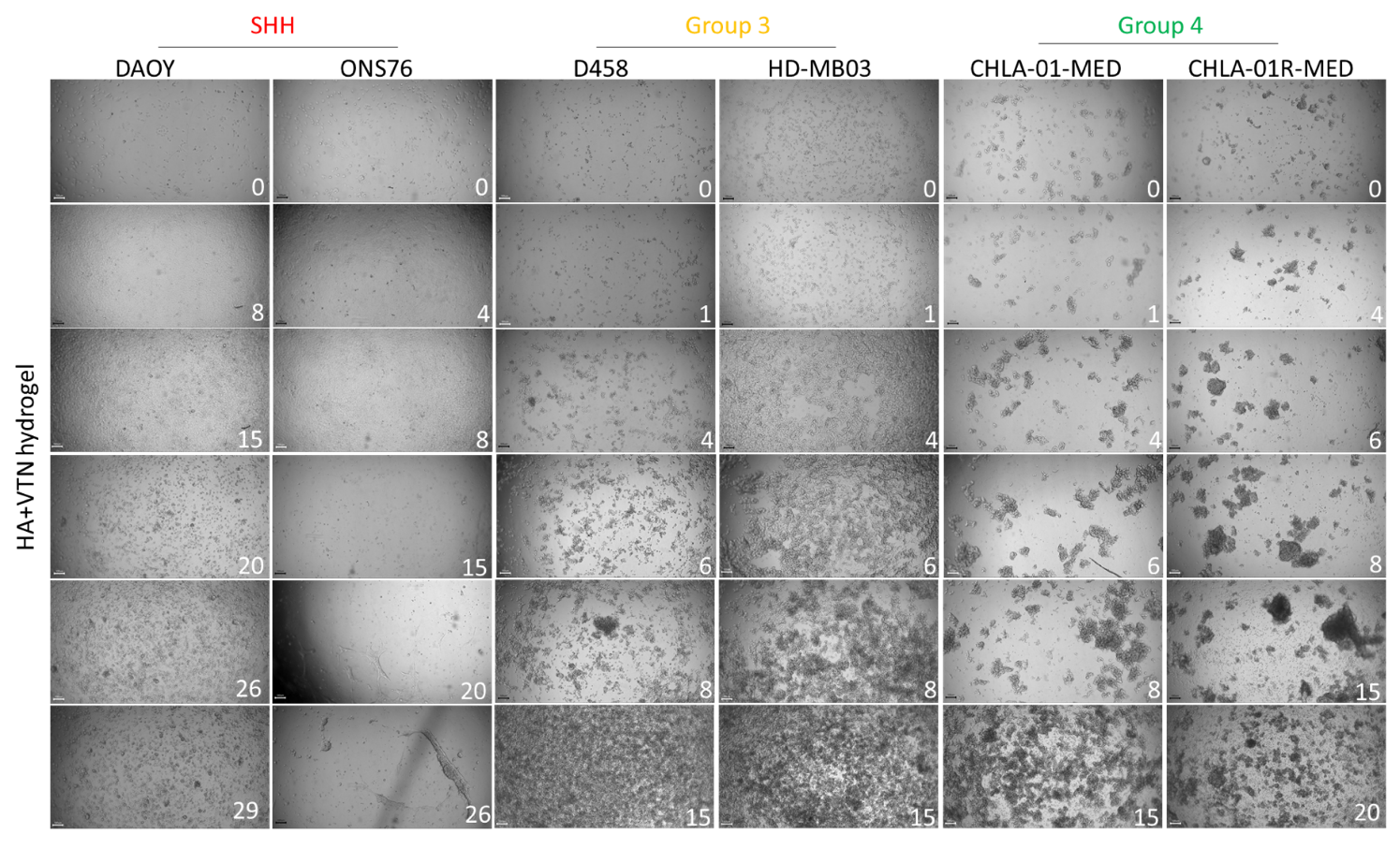


**Figure S10.** **Cell growth on vitronectin-supplemented HA hydrogels revealed remarkable differences between SHH cell lines and Group 3 and Group 4 cell lines.** Growth patterns were monitored over time (number in the bottom right corner equals day of growth). SHH cell lines (DAOY, ONS76) immediately attached and spread as a very thin monolayer on top of the vitronectin-supplemented HA hydrogels. This monolayer appeared very flat and tight with no single-cell borders being visible. After 2 weeks, the cell layer became overgrown and some cells/cell patches floated off. Group 3 (D458, HD-MB03) and Group 4 cell lines (CHLA-01-MED, CHLA-01R-MED) behaved similarily as on the laminin-supplemented HA hydrogels. Group 3 cell lines adhered as single cells across the entire gel area and filled the gaps over time with a laminar coating, while Group 4 cell lines started growing as well-defined patches and filled up the space over time (scale bar = 100 µm).


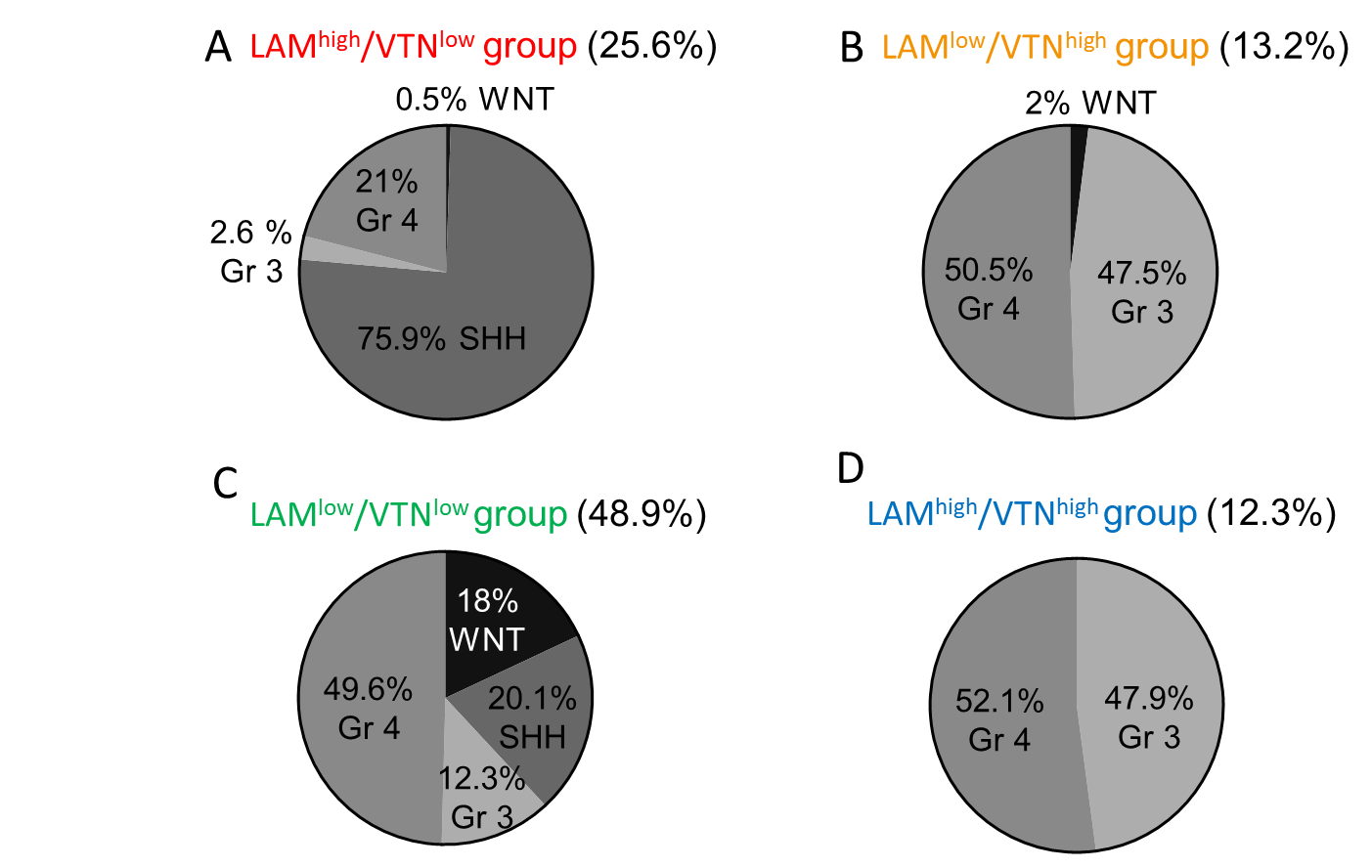


**Figure S11. ECM subtypes are composed of different MB subgroup proportions.** (A) The ‘LAM^high^/VTN^low^’ group mainly comprised SHH and Group 4 cases, while (B) the ‘LAM^low^/VTN^high^’ group was composed of mainly Group 3 and Group 4 cases. (C) Cases that expressed low levels of both laminin and vitronectin were found in all subgroups, while (D) high-level expression of both markers was observed explicitly in Group 3 and Group 4 patients.

**Table S1.** Clinicopathological characteristics of MB patients included in the TMAs

|  | SHH | Group 3 | Group 4 |
| --- | --- | --- | --- |
| Gender  F  M  Unknown | 3  5  3 | 0  6  0 | 6  14  5 |
| Age, years  <3  ≥3  Unknown | 0  6  5 | 2  4  0 | 3  18  4 |
| Metastatic status  M−  M+  Unknown | 5  4  2 | 4  1  1 | 9  13  3 |
| Treatment  CT  RT  CT + RT  Unknown | 1  1  3  6 | 0  1  5  0 | 2  0  15  8 |
| Status  A  D  Unknown | 2  4  5 | 2  3  1 | 6  10  9 |
| M = male; F = female; CT = chemotherapy; RT = radiotherapy; A = alive; D = dead. | | | |
